# Germinal Center–Like Tertiary Lymphoid Structures Mark Immune Responsiveness and Enable Checkpoint Immunotherapy in Bladder Cancer

**DOI:** 10.32604/or.2026.077808

**Published:** 2026-06-16

**Authors:** Zhihao Yin, Xi Zhen, Haonan Li, Haiqiang Duan, Xiaowei Hu, Tianxi Yu, Qing Shi, Ziyi Liu, Yaowei Li, Peng Zhang, Peng Dai, Meihui Zhao, Ziqi Wang, Changfu Li, Di Wang, Zhichao Tong

**Affiliations:** 1 NHC Key Laboratory of Molecular Probe and Targeted Theranostics, Harbin Medical University Cancer Hospital, Harbin Medical University, Harbin, China; 2 Department of Urology, Harbin Medical University Cancer Hospital, Harbin, China; 3 Heilongjiang Provincial Key Laboratory of Basic Medical Sciences in Urology Cancer, Harbin Medical University Cancer Hospital, Harbin, China; 4 College of Life Sciences, Northeast Forestry University, Harbin, China; 5 Department of Urogenital Medical Oncology, Harbin Medical University Cancer Hospital, Harbin, China; 6 Department of Breast Surgery, Harbin Medical University Cancer Hospital, Harbin, China; 7 Department of Cystoscope Center, Harbin Medical University Cancer Hospital, Harbin, China; 8 Biobank, Harbin Medical University Cancer Hospital, Harbin, China; 9 Department of Urology, Klinikum Rechts der Isar, Technical University of Munich, Munich, Germany

**Keywords:** Tertiary lymphoid structures, bladder cancer, germinal center (GC)–like tertiary lymphoid structure, tumor microenvironment, programmed death-ligand 1

## Abstract

**Backgrounds:** Tertiary lymphoid structures (TLSs) are increasingly recognized as modulators of anti-tumor immunity, yet their clinical relevance in bladder cancer remains incompletely understood, partly owing to heterogeneity in their maturation states. Here, we demonstrate that germinal center (GC)–like TLS maturity, rather than TLS presence alone, is closely associated with immune activation and therapeutic response to Programmed Death-Ligand 1 (PD-L1) blockade in bladder cancer. The objective of this study was to systematically investigate the clinical significance, biological function, and therapeutic potential of tertiary lymphoid structure (TLS) maturation in bladder cancer. Specifically, we aimed to determine whether GC-like TLS maturity provides prognostic and predictive value beyond TLS presence alone, to elucidate the immune programs and tumor microenvironment remodeling associated with TLS maturation, and to explore whether TLS maturation can be therapeutically induced to enhance responsiveness to PD-L1 blockade. **Methods:** We performed an integrative analysis combining multi-cohort transcriptomics, spatially resolved histopathology, single-cell RNA sequencing, and functional murine experiments. TLS maturation states were defined using gene-expression–based GC-like TLS signatures and validated through multiplex immunohistochemistry. Clinical relevance was assessed in public immunotherapy cohorts and an independent neoadjuvant PD-L1–treated muscle-invasive bladder cancer (MIBC) cohort. Tumor immune microenvironment remodeling and chemokine-mediated cellular crosstalk were analyzed using deconvolution, Weighted Gene Co-expression Network Analysis (WGCNA), and CellChat. The therapeutic inducibility of TLS maturation was examined using a lymphotoxin-β receptor (LTβR) agonist in combination with PD-L1 blockade in a syngeneic bladder cancer model. **Results:** Across multiple transcriptomic cohorts, tumors enriched for GC-like TLS signatures exhibited significantly prolonged survival and higher objective response rates to anti–PD-L1 therapy, whereas less mature TLS phenotypes showed no consistent association with clinical association. These observations were independently validated in a neoadjuvant PD-L1–treated muscle-invasive bladder cancer cohort, in which high mature TLS density was associated with major pathological response and prolonged event-free survival, outperforming PD-L1 expression. Integrative histopathological and transcriptomic analyses indicated that GC formation marks a functional transition linking humoral immune programs with cytotoxic effector activity and shaping a memory-prone, pro-inflammatory tumor immune microenvironment. Chemokine signaling via the CC chemokine ligand 21 (CCL21)–C-C chemokine receptor type 7 (CCR7) and C-X-C motif chemokine ligand 12 (CXCL12)–C-X-C chemokine receptor type 4 (CXCR4) axes was strongly associated with TLS maturation and spatial organization. Finally, in a syngeneic bladder cancer model, pharmacological activation of lymphotoxin-β receptor signaling promoted TLS maturation and enhanced the antitumor efficacy of PD-L1 blockade. **Conclusions:** Together, these findings suggest that GC-like TLS maturity represents a clinically relevant biomarker and a potential therapeutic entry point for precision immunotherapy in bladder cancer. Therapeutic strategies that promote TLS maturation may convert immune-cold tumors into checkpoint-responsive states, providing a mechanistically grounded precision immunotherapy approach.

## 1 Introduction

Bladder cancer is among the most prevalent malignancies of the urinary tract, with muscle-invasive bladder cancer (MIBC) representing its most aggressive clinical phenotype, characterized by high recurrence rates, early metastatic dissemination, and poor survival outcomes. Although immune checkpoint inhibitors (ICIs), particularly those targeting the programmed death-1/programmed death-ligand 1 (PD-1/PD-L1) axis, have reshaped the therapeutic landscape for advanced bladder cancer, durable clinical benefit remains restricted to a minority of patients, with objective response rates (ORRs) consistently ranging between 20–30% [1,2]. This profound inter-patient heterogeneity highlights a central unmet need in uro-oncology: the lack of robust biomarkers that accurately predict response to ICI therapy. Consequently, deciphering the key immunological determinants within the tumor immune microenvironment (TIME) that govern sensitivity or resistance to ICIs has become a major research priority [3].

In this context, tertiary lymphoid structures (TLSs)—ectopic lymphoid aggregates that arise *de novo* at sites of chronic inflammation and cancer—have emerged as critical regulators of anti-tumor immunity [4,5]. Across multiple solid tumor types, including melanoma and non–small cell lung cancer, the presence of TLSs has been associated with improved prognosis and enhanced responsiveness to ICIs, positioning them as potential intratumoral hubs for local adaptive immune priming [6,7]. Importantly, TLSs are not uniform entities but instead exist along a maturation continuum, ranging from loose lymphocytic aggregates to highly organized secondary follicles containing active germinal centers (GCs). Accumulating evidence increasingly suggests that TLS maturity, rather than TLS presence alone, determines their immunological competence, with GC-containing TLSs supporting antigen presentation, B cell affinity maturation, and sustained T cell activation [8,9].

However, in bladder cancer, the clinical and biological significance of TLS maturation has not been systematically delineated. In particular, it remains unclear whether GC-like TLSs confer prognostic and predictive value beyond established immune biomarkers, how TLS architectural maturation is linked to coordinated adaptive immune programs within the tumor immune microenvironment (TIME), and whether TLS maturation can be therapeutically leveraged to enhance responsiveness to immune checkpoint blockade [7].

Here, by integrating transcriptomic profiling, histopathological evaluation, spatial analysis, and functional experimentation across multiple cohorts, we aimed to develop a unified framework to investigate the relationship between TLS maturation state and clinical outcome, immune function, and therapeutic responsiveness in bladder cancer. Specifically, we sought to determine whether GC-like TLS maturity could serve as a clinically relevant discriminator in immunotherapy cohorts and whether this concept could be translated into a practical pathology-based metric in a neoadjuvant PD-L1–treated MIBC cohort. We further aimed to explore the immune circuitry associated with germinal center formation, its impact on tumor immune microenvironment remodeling, the chemokine-mediated cellular crosstalk underlying TLS maturation, and the potential therapeutic inducibility of TLS maturation to enhance responsiveness to PD-L1 blockade. Ultimately, this study was designed to evaluate whether GC-like TLS maturity may represent both a predictive biomarker and a potential therapeutic target for precision immunotherapy in bladder cancer [5,10,11].

## 2 Materials and Methods

### 2.1 Immunotherapy Cohort Data Collection

This study integrated multi-cohort transcriptomic analyses, a institutional neoadjuvant immunotherapy cohort, and N-butyl-N-(4-hydroxybutyl) nitrosamine (BBN)-induced spontaneous murine models of bladder cancer to investigate the clinical relevance, biological function, and therapeutic inducibility of tertiary lymphoid structure (TLS) maturation.

IMvigor210 is a clinical cohort study designed to investigate the application of anti-PD-L1 therapy in patients with metastatic urothelial carcinoma (mUC) [12,13]. Data of bladder cancer patients were obtained from the R package “IMvigor210CoreBiologies”, and 195 samples complete expression and clinical annotation were retained after screening.

For the institutional cohort, formalin-fixed paraffin-embedded (FFPE) pretreatment tumor resection specimens (n = 60) were obtained from patients with muscle-invasive bladder cancer who underwent surgical treatment at Harbin Medical University Cancer Hospital. Tumor specimens were collected prior to systemic therapy and were used for histopathological evaluation, immunohistochemistry, and multiplex immunofluorescence analyses of TLS-related markers.

All patients from our institution in this cohort received PD-L1 blockade as neoadjuvant therapy, representing a first-line systemic immunotherapeutic approach in the perioperative setting. Prior intravesical therapy was permitted; a subset of patients had previously received bacillus Calmette–Guérin (BCG) for non–muscle-invasive disease, whereas none had received prior systemic immune checkpoint inhibitors. Patients with a history of systemic chemotherapy for metastatic disease were excluded.

Patients were excluded if they met any of the following criteria: (1) history of systemic chemotherapy for metastatic disease (2) previous treatment with immune checkpoint inhibitors (3) evidence of distant metastasis at baseline (4) active autoimmune disease requiring systemic therapy or ongoing immunosuppressive treatment (5) uncontrolled infection or severe comorbid conditions that could interfere with treatment (6) incomplete clinical information or loss to follow-up.

Patients were eligible for inclusion if they met the following criteria: (1) histologically confirmed muscle-invasive urothelial carcinoma; (2) clinical stage T2–T4a, N0–N2, M0; (3) Eastern Cooperative Oncology Group (ECOG) performance status 0–1; (4) adequate hematologic, hepatic, and renal function; and (5) absence of active autoimmune disease or ongoing immunosuppressive therapy. These criteria ensured that patients were suitable candidates for perioperative immunotherapy and radical cystectomy.

### 2.2 Non-Immunotherapy Single-Cell Data Collection

Single-cell data of bladder cancer patients were downloaded from the Gene Expression Omnibus (GEO, https://www.ncbi.nlm.nih.gov/gds), including GSE129845 (human bladder samples were filtered and retained), GSE145137 (only single-cell data from patients), GSE222315, GSE267718 (tumor cells from patients were filtered and retained), and GSE149652 (CD4^+^ and CD8^+^ T cells from untreated patients). Standard quality-control filtering and annotation provided in the original studies were adopted.

### 2.3 TLS Signature Scoring

To quantify the maturation status of TLS in bulk RNA-seq data, we performed gene set scoring on the bulk RNA-seq expression matrix, referencing three TLS-related gene signatures (including GC-like TLS, primary follicle-like TLS, and early TLS) curated from previously published studies [14,15,16].

GC-like TLS: CD19 (Cluster of Differentiation 19 molecule), BCL6 (B-cell lymphoma 6 transcription repressor), AICDA (activation-induced cytidine deaminase), MKI67 (marker of proliferation Ki-67), CXCL13 (C-X-C motif chemokine ligand 13), CD40LG (CD40 ligand), IL21 (interleukin 21), CXCR5 (C-X-C motif chemokine receptor 5), FCER2 (Fc fragment of IgE receptor II, CD23), and CR2 (complement receptor 2, CD21).

Primary follicle-like TLS: MS4A1 (membrane-spanning 4-domains subfamily A member 1, CD20), CD79A (CD79a molecule), CXCL13 (C-X-C motif chemokine ligand 13), CD21 (complement receptor 2, CR2), LTB (lymphotoxin beta).

Early TLS: CXCR5 (C-X-C motif chemokine receptor 5), CCL19 (CC chemokine ligand 19), CCL21 (CC chemokine ligand 21), CD3E (CD3E molecule), CD4 (CD4 molecule), CD8 (CD8 molecule), CCR7 (C-C chemokine receptor type 7).

Samples were stratified into mature TLS and immature TLS groups based on the median value of the “GC-like TLS score”.

### 2.4 Survival Analysis

The Kaplan-Meier method was used to evaluate the impact of each variable on survival time. Survival outcomes, including overall survival (OS) and event-free survival (EFS), were estimated using the Kaplan-Meier method, and between-group survival differences were compared via the two-sided log-rank test. The optimal cutoff value for continuous variables was determined using the “survminer” R package (0.5.1 version) [17], and these variables were converted into binary variables for more precise analysis. Additionally, multivariate Cox regression analyses were conducted to evaluated the impact of multiple variables simultaneously, thereby identifying independent prognostic factors for survival. Univariate and multivariate Cox proportional hazards regression models were sequentially constructed to identify independent prognostic factors for the above survival endpoints. Variables with a *p*-value < 0.1 in the univariate Cox analysis, along with pre-specified variables of clinical significance, were entered into the multivariate Cox regression model. All regression tests were two-sided, with a *p*-value < 0.05 considered statistically significant. The proportional hazards (PH) assumption of the final Cox model was verified using Schoenfeld residuals, with no violation of the PH assumption detected. Hazard ratios (HRs) with corresponding 95% confidence intervals (CIs) were calculated to quantify the independent prognostic association between each variable and survival outcomes, and the results of the multivariate Cox regression were visualized using a forest plot.

### 2.5 Evaluation of TLS Maturation Status and Tumor Microenvironment at the Bulk RNA-Seq Level

To characterize the relationship between the maturation status of TLS and the characteristics of the tumor immune microenvironment (TIME), two complementary analyses were performed on the normalized bulk RNA-seq expression matrix. First, the estimation of stromal and immune cells in malignant tumor tissues using expression data (ESTIMATE) algorithm was applied to calculate the ImmuneScore for each sample, which served to quantify the overall immune level of the sample and provide a macroscopic context for subsequent functional comparisons [18]. Second, to investigate the activity of immune functional pathways directly related to TLS maturation, gene sets associated with immunity and lymphoid structure formation were curated from the Molecular Signatures Database (MSigDB, https://www.gsea-msigdb.org/gsea/msigdb); these included gene sets involved in antigen processing and presentation, chemokine/chemokine receptor signaling, IFN-γ response, and innate immune pathways from the Hallmark, Gene Ontology (GO), and Reactome databases. Based on the aforementioned normalized expression matrix, the Gene Set Variation Analysis (GSVA) algorithm was applied to compute the pathway enrichment score (GSVA score) for each sample. Differences in ESTIMATE scores and GSVA scores between the mature TLS group and immature TLS group were evaluated using the two-sided Wilcoxon rank-sum test, respectively [19].

### 2.6 Cytolytic and Humoral Immunity Scoring

To quantify specific immune functional states, we calculated two distinct scores using Gene Set Variation Analysis (GSVA) with the R package “GSVA” (2.3.1 version).

Cytolytic activity score: To assess the effector functions of cytotoxic T cells and natural killer (NK) cells, we quantified the enrichment of a cytolytic signature comprising two canonical effector genes: Granzyme A (GZMA) and Perforin 1 (PRF1). This approach adapts the methodology described in published literature [20].

Germinal center-humoral immunity activation score (GC-humoral score): To reflect gene expression programs associated with B cell activation, germinal center reactions, and antibody affinity maturation, we constructed a specific gene set consisting of seven key markers [14,15,16]: AICDA and BCL6 (GC reaction markers); MZB1, XBP1, and PRDM1 (plasma cell differentiation and antibody secretion regulators); and IGHG1 and IGHA1 (immunoglobulin heavy chains). The GSVA score for this gene set was calculated to represent the overall intensity of the humoral immune response in the tumor microenvironment.

Statistical correlations between the GC-like TLS score and these functional scores were subsequently evaluated using Spearman’s rank correlation analysis.

### 2.7 Bulk RNA-Seq Deconvolution

To delineate the systemic immunomodulatory impact of GC-like tertiary lymphoid structures on the tumor microenvironment (TME), we first performed bulk RNA-seq deconvolution to characterize the overall differences in TME cellular composition between TLS subgroups; the deconvolution results were further validated and refined at single-cell resolution via subsequent scRNA-seq data analysis.

To assess differences in the composition of cell subtypes between the mature and immature TLS groups, transcriptome deconvolution was performed on the transcripts per million (TPM)-normalized expression matrix using Cell-type Identification By Estimating Relative Subsets of RNA Transcripts (CIBERSORT) [21,22]. For CIBERSORT analysis, the LM22 immune cell reference matrix was adopted with 1000 permutations performed to generate relative cell fractions. Differences in the relative abundance scores of cell subtypes between the two groups (TLS_mature vs. TLS_immature) were compared using the two-sided Wilcoxon rank-sum test.

### 2.8 Identification of Differentially Expressed Genes

Differential expression analysis was performed on the integer count matrix using the R package “DESeq2” (1.49.3 version) [23,24]. To improve test sensitivity and eliminate low-expression noise, genes were preliminarily filtered using the transcripts per million matrix: only genes with TPM > 1 in at least 10% of samples and expressed in at least 20% of samples in either comparison group were retained. This subset of genes was then applied to the count matrix to construct the DESeq2 data object. The screening thresholds for differentially expressed genes (DEGs) were |log_2_FoldChange| > 1 and adjusted *p*-value (padj) < 0.05. Upregulated genes (log_2_FC > 1 and padj < 0.05) were used for subsequent Gene Ontology (GO) and Kyoto Encyclopedia of Genes and Genomes (KEGG) enrichment analyses.

### 2.9 GO and KEGG Enrichment Analysis

For the DEGs significantly upregulated in the mature TLS group, Gene Ontology (GO) biological process (BP) and KEGG pathway enrichment analyses were conducted using the R-based “clusterProfiler” package (4.18.2 version) [25]. The enrichGO and enrichKEGG functions were used to perform over-representation analysis (ORA), with the Benjamini–Hochberg method applied to correct for multiple testing. Adjusted *p*-values < 0.05 were considered statistically significant. The top 15 most significantly enriched pathways were visualized as dot plots.

### 2.10 Construction of WGCNA, Module-Trait Correlation, and Hub Gene Enrichment Analysis

A weighted gene co-expression network analysis (WGCNA) was constructed based on the log fold changed TPM expression matrix using the R package “WGCNA” (1.73 version) [26]. The following steps were implemented: an optimal soft-thresholding power was selected to construct a scale-free network; the weighted adjacency matrix and topological overlap matrix (TOM) were calculated; hierarchical clustering was performed based on TOM dissimilarity, and dynamic tree cut was applied to identify modules, followed by merging of similar modules (mergeCutHeight = 0.25). The module eigengene (ME) of each module was computed and subjected to correlation analysis with the clinical phenotype (TLS_mature binary variable), yielding a module-trait relationship matrix. Significant modules positively correlated with TLS_mature (correlation coefficient ≥ 0.3 and *p* < 0.05) were selected as candidate modules.

Hub genes within the candidate modules were identified using intramodular connectivity and module membership metrics: module membership (MM, defined as the Pearson correlation between individual gene expression and the corresponding ME) and gene significance (GS, defined as the correlation between individual gene expression and TLS_mature) were calculated; genes satisfying |MM| ≥ 0.7 and |GS| ≥ 0.2 were defined as hub genes. Gene Ontology Biological Process (GO BP) and KEGG pathway enrichment analyses were conducted on these hub genes, using the same methodology as the differential gene enrichment analysis. *p*-values were corrected for multiple testing using the Benjamini–Hochberg method, and a false discovery rate (FDR) < 0.05 was considered statistically significant.

### 2.11 scRNA-Seq Data Analysis

This single-cell analysis was performed to validate the TME cellular composition differences identified by the aforementioned bulk RNA-seq deconvolution, and further clarify the functional orientation of TME cell populations modulated by GC-like TLS, so as to fully delineate its systemic immunomodulatory effect.

The downloaded single-cell datasets were subjected to data normalization using the Python-based “Scanpy” package (1.11.1 version): first, total count normalization was performed via the “sc.pp.normalize” function, followed by data scaling using the “sc.pp.scale” function to minimize the impact of technical noise [27]. Batch effects were corrected using the Harmony algorithm [28]. Based on the normalized and batch-effect-corrected data, linear dimensionality reduction was conducted through principal component analysis (PCA); subsequently, a cell neighbor graph was constructed with the “sc.pp.neighbors” function, and unsupervised clustering analysis was executed using the “sc.tl.leiden” function. The clustering results were visualized via the Uniform Manifold Approximation and Projection (UMAP) algorithm.

To identify immune and stromal cell populations, cell gene set scores (score_genes) were calculated based on the classic immune/stromal marker gene sets curated from published literature [29,30], and candidate sets of immune and stromal cells were screened out. Refined analysis was performed on these candidate subsets: highly variable genes were re-identified on the subsets, followed by PCA implementation, adjacency graph construction, and high-resolution clustering. Differential gene expression analysis was carried out using the “sc.tl.rank_genes_groups” function, and annotation of each cell cluster was completed by integrating classic cell type marker genes and characteristic marker genes reported in published literature.

### 2.12 Cell-Cell Communication Analysis via CellChat Based on Intersection Genes at the Single-Cell Level

To identify the enrichment of TLS maturation-related genes in single-cell subtypes and infer cell-cell communication among these subtypes, we first obtained an intersection gene set at the bulk level by taking the intersection of significantly upregulated differentially expressed genes (DEGs) in the “TLS_mature” group and hub genes of modules positively correlated with mature TLS in WGCNA. This gene set was mapped to the single-cell expression matrix, and the intersection gene score for each cell cluster was calculated on the single-cell data. Clusters with scores > 0 were selected based on the distribution of this score across cell clusters, and these cell subtypes were designated as candidate cell populations for “CellChat” analysis (2.1.2 version) [31,32].

Subsequently, a CellChat object was constructed for the selected cell populations to infer ligand-receptor communication networks: log-normalized counts were used as input, the human ligand-receptor database was specified [33], and the standard CellChat workflow was run to calculate communication probabilities between cell populations and summarize pathway-level networks. To assess statistical significance, the randomization test provided by CellChat (n = 1000 permutations) was used to estimate the significance of interactions, and *p*-values were corrected for multiple testing using the Benjamini–Hochberg method (with a false discovery rate (FDR) < 0.05 set as the significance threshold).

### 2.13 Histology and Immunohistochemistry (IHC)

Human tumor samples were obtained from patients with muscle-invasive bladder cancer who received treatment at our institution, with approval from the institutional ethics committee. The study was approved by the Medical Ethics Committee of Harbin Medical University Cancer Hospital (Approval No. KY2024-100), and written informed consent was obtained from all participants. Formalin-fixed, paraffin-embedded (FFPE) tissue sections (4 μm) were stained with hematoxylin and eosin (H&E) (Solarbio, Beijing, China) for morphological assessment. Paraffin sections were dewaxed with xylene (FuYu, Tianjin, China) and gradient ethanol (TianLi, Tianjin, China), followed by antigen retrieval using EDTA antigen retrieval buffer (Beyotime, Shanghai, China, Cat# P0085). Subsequently, endogenous peroxidase was inactivated with 3% hydrogen peroxide. After blocking with immunofluorescence blocking buffer (Beyotime, Shanghai, China, Cat# P0260), the sections were incubated with primary antibodies overnight at 4°C. Following 1-h incubation with secondary antibodies (goat anti-rabbit or anti-mouse IgG), the paraffin sections were stained with DAB Detection Kit (Zhong Shan-Golden Bridge, Beijing, China, Cat# ZLI-9019) and counterstained with hematoxylin (Solarbio, Beijing, China). Immunohistochemistry (IHC) was performed using antibodies against CD20, CD3, CD21, CD23, and Ki67. The following antibodies were used for IHC: CD20 (1:500; Abcam, Cambridge, UK; Cat. No. ab64088), CD3 (1:200; Abcam, Cambridge, UK; Cat. No. ab16669), CD21 (1:500; Abcam, Cambridge, UK; Cat. No. ab75985), CD23 (1:200; Abcam, Cambridge, UK; Cat. No. ab16702), Ki67 (1:200; Abcam, Cambridge, UK; Cat. No. ab1667), anti-rabbit IgG (1:200; Proteintech, Rosemont, IL, USA; Cat. No. SA00001-2), anti-mouse IgG (1:200; Proteintech, Rosemont, IL, USA; Cat. No. SA00001-1). TLS density was quantified as the number of GC-like TLSs per square millimeter of viable tumor area. Patients were dichotomized into high- and low-density groups using the cohort median. All histopathological evaluations were independently performed by two experienced pathologists blinded to clinical outcomes.

### 2.14 Immunofluorescence (IF)

Multiplex immunofluorescence staining was performed using the Pano Six-Color Immunofluorescence Kit (Panovue, Beijing, China, Cat# 0004100100). Following dehydration with xylene (FuYu, Tianjin, China) and rehydration with graded ethanol (TianLi, Tianjin, China), sections were immersed in Tris-EDTA antigen retrieval buffer (Beyotime, Shanghai, China) (pH 8.0) and subjected to antigen retrieval via microwave heating. Sections were allowed to cool to room temperature. To block endogenous antigens, sections were treated with 1% bovine serum albumin (BSA) (Solarbio, Beijing, China) at room temperature for 30 min. Primary antibodies were incubated at room temperature for 1 h, followed by incubation with secondary antibodies at room temperature for 30 min. Tyrosinase signal amplification (TSA) was performed using fluorescent reagents (PPD 520, PPD 570, PPD 620, PPD 650, and PPD 780; Panoue, Beijing, China; 1:100 dilution) for 10 min at room temperature. Following staining for all targets, nuclei were stained with DAPI (1:100, Panowei, Beijing, China) for 15 min at room temperature. After each step, sections were washed three times with Tris-Buffered Saline with Tween-20 (TBST) solution for 2 min each. Finally, sections were mounted with neutral binder. Images were acquired using a confocal laser scanning microscope (Axio Imager Z2, Carl Zeiss Microscopy GmbH, Jena, Germany). All immunofluorescence assays were independently replicated at least three times.

For multiplex immunofluorescence staining, primary antibodies included CXCL12 (1:200; Abcam, Cambridge, UK; Cat# ab9797), CD20 (1:200; Abcam, Cambridge, UK; Cat. No. ab64088), CD21 (1:200; Abcam, Cambridge, UK; Cat. No. ab75985), CD23 (1:200; Abcam, Cambridge, UK; Cat. No. ab16702).

### 2.15 BBN-Induced Bladder Cancer Model

Eight-week-old male C57BL/6 mice (20–22 g) (Changsheng Biotechnology, Benxi, Liaoning, China) were used to establish a chemically induced bladder cancer model. Mice were kept in individually ventilated cages with autoclaved bedding, with a 12-h light–dark cycle, controlled temperature (25°C) and humidity (30%–70%), and ad libitum access to food and autoclaved water.

Bladder tumorigenesis was initiated by administering N-butyl-N-(4-hydroxybutyl) nitrosamine BBN (MedChemExpress, Monmouth Junction, NJ, USA; Cat. No. HY-W755252) at a concentration of 0.1% (w/v) in drinking water for 12 consecutive weeks. Histopathological evaluation using hematoxylin and eosin (H&E) staining was performed at weeks 4, 8, and 12 during BBN exposure to monitor the progression of bladder lesions and confirm tumor development.

Following the completion of BBN administration, mice with histologically confirmed bladder tumors were enrolled in therapeutic studies. Starting at week 13, tumor-bearing mice were randomly assigned to four treatment groups (n = 8 per group): (i) vehicle control, (ii) anti–programmed death-ligand 1 (PD-L1) antibody monotherapy (clone 10F.9G2; Bio X Cell, West Lebanon, NH, USA, Cat# BE0101), (iii) lymphotoxin-β receptor (LTβR) agonist monotherapy (iv) combination therapy (anti–PD-L1 + LTβR agonist). The LTβR agonist antibody (clone 5G11b; Bio-Rad Laboratories, Hercules, CA, USA, Cat# MCA2244) was administered via intraperitoneal injection at a dose of 100 μg per mouse once weekly for three weeks (total treatment duration: 21 days). The anti–PD-L1 antibody was administered intraperitoneally at 100 μg per mouse on the same schedule. Mice in the combination group received both agents according to the above regimen. The three-week treatment schedule was selected based on previously reported murine immunotherapy protocols using anti–PD-L1 antibodies, which typically involve repeated administration over a 2–3 week period to allow sufficient immune activation and antitumor responses [34,35].

Animals were randomized prior to treatment initiation, and investigators performing outcome assessments were blinded to group allocation. Endpoints, including tumor burden and histopathological evaluation, were assessed at the completion of the treatment period.

All animal procedures were approved by the Institutional Animal Care and Use Committee of Harbin Medical University (Approval No. GJZDYF2024-001) and conducted in accordance with institutional guidelines and the ARRIVE recommendations.

### 2.16 Treatment Response Assessment

Radiologic and pathologic responses were evaluated sequentially to characterize treatment efficacy following neoadjuvant PD-1 blockade.

Radiologic tumor response during immunotherapy was assessed using immune Response Evaluation Criteria in Solid Tumors (iRECIST, version 1.1). Baseline imaging was performed prior to treatment initiation, and follow-up computed tomography (CT) or magnetic resonance imaging (MRI) was conducted every two treatment cycles. Responses were classified as immune complete response (irCR), immune partial response (irPR), immune stable disease (irSD), or immune progressive disease (irPD). Immune progressive disease required confirmation on subsequent imaging according to iRECIST guidelines. The immune objective response rate (irORR) was defined as the proportion of patients achieving irCR or irPR.

Following completion of neoadjuvant therapy, all patients underwent radical cystectomy with pelvic lymph node dissection. Surgical specimens were evaluated by dedicated genitourinary pathologists who were blinded to imaging outcomes. Post-treatment pathological stage was determined according to the AJCC TNM staging system (ypTNM). Pathologic complete response (pCR) was defined as the absence of residual invasive tumor in both the primary tumor bed and regional lymph nodes (ypT0N0). Major pathological response (MPR) was defined as ≤10% residual viable tumor cells within the tumor bed. Residual tumor percentage was estimated on hematoxylin–eosin–stained sections.

Radiologic response assessed by iRECIST does not necessarily correspond to pathological response, and therefore both imaging-based and pathology-based endpoints were analyzed independently.

### 2.17 Image Acquisition and Spatial Validation

Whole-slide images were acquired using a high-resolution digital pathology scanner (GScan-12, G Cell Technology, Guangzhou, China). For spatial validation of chemokine signaling, multiplex immunofluorescence staining was performed using antibodies against CD20, CD21, CD23, CXCL12 (Abcam, Cambridge, UK; Cat# ab9797; dilution 1:200), and DAPI nuclear counterstain. Spatial co-localization of CXCL12 with B-cell–rich regions was qualitatively assessed. Image analysis was performed using ImageJ software (version 1.53, National Institutes of Health, Bethesda, MD, USA).

### 2.18 Statistical Analysis

All statistical analyses were performed using R software (version 4.5.1). All statistical tests were two-sided, and a *p*-value < 0.05 was considered statistically significant for unadjusted comparisons. The Wilcoxon rank-sum test was adopted for comparisons between two groups in the study, while the chi-square test or Fisher’s exact test was used for the comparison of categorical variables, and Pearson’s correlation coefficient was applied for correlation analysis. *P*-values from all multiple comparisons were corrected using the Benjamini–Hochberg method, and a false discovery rate (FDR) < 0.05 after correction was considered statistically significant.

## 3 Results

### 3.1 GC-Like TLS Maturation as a Biomarker of Survival Benefit and Immunotherapy Response in Bladder Cancer

**Figure 1:**
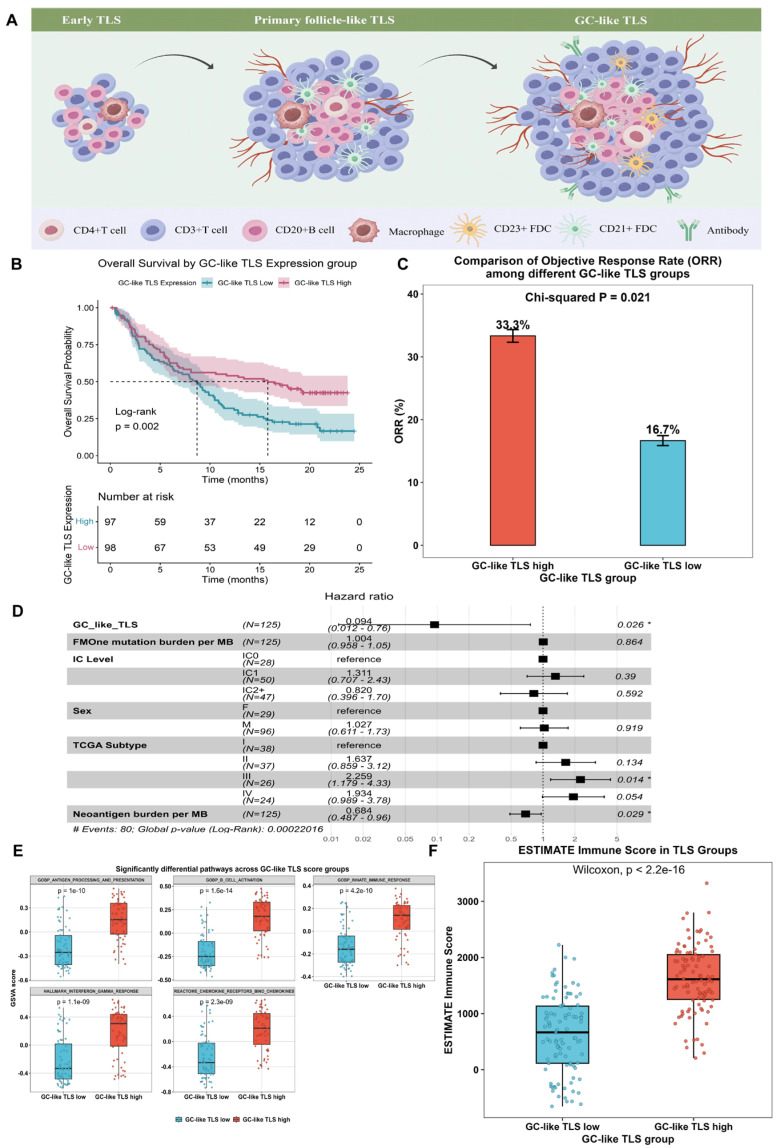
**Association Analysis of GC-like TLS Expression Levels with Survival, Clinical Efficacy, and Immune Characteristics in Patients.** (**A**) Schematic illustration of tertiary lymphoid structure (TLS) formation and maturation. (**B**) Kaplan–Meier curves of overall survival stratified by GC-like TLS expression (95% CIs: shaded areas). (**C**) Bar chart of objective response rate (ORR) across different GC-like TLS groups. Error bars = 95% CI. (**D**) Forest plot of multivariate Cox regression for overall survival: HR (squares), 95% CIs (horizontal lines), HR = 1 (vertical dashed line). *indicates statistical significance with *p* < 0.05. The proportional hazards (PH) assumption of Cox regression models was verified using Schoenfeld residuals, and no significant violations were detected. (**E**) GSVA scores of significantly enriched pathways between GC-like TLS high- and low-expression groups (*p* < 0.05). (**F**) ESTIMATE ImmuneScore comparison between different GC-like TLS expression groups. CD: Cluster of Differentiation; FDC: Follicular Dendritic Cell; CI: Confidence Interval; HR: Harzard ratio; IC: Immune Cell; TCGA: The Cancer Genome Atlas Subtype; FMOne: FoundationOne; MB: Megabase.

Transcriptomic stratification of tertiary lymphoid structures (TLS) based on their maturation stage robustly identified the germinal center-like (GC-like) TLS phenotype as an independent determinant of favorable clinical outcomes in bladder cancer [7]. Our analysis defined three distinct TLS subsets—early (E-TLS), primary follicle-like (PFL-TLS), and secondary follicle/GC-like TLS—across multiple cohorts (Fig. 1A) (Table 1). This three-tier framework is aligned with the prevailing concept that germinal center formation represents the key maturation inflection point that confers functional competence [36].

This stratification revealed that a high GC-like TLS transcriptional signature was significantly associated with prolonged overall survival (Log-rank *p* = 0.002), whereas no significant survival association was observed for less mature TLS phenotypes (Fig. 1B). Within the context of immune checkpoint blockade, analysis of the IMvigor210 cohort demonstrated that patients with high GC-like TLS signature tumors achieved a markedly superior objective response rate to anti-PD-L1 therapy (33.3% vs. 16.7%, Chi-square *p* = 0.021) (Fig. 1C). Multivariate Cox regression confirmed the independent predictive power of this signature, as it remained a significant protective factor (Hazard Ratio < 1, *p* = 0.026) after rigorous adjustment for tumor Immune Cell (IC) level, sex, and clinical stage (Fig. 1D).

The biological relevance of this association was corroborated by pathway enrichment analysis (Fig. 1E), which showed concomitant activation of key adaptive immune pathways related to B cell activation and antigen presentation in GC-like TLS-high tumors (Fig. 1F). Notably, this enriches the emerging view that TLS-associated benefit is not simply “more lymphocytes,” but a distinct immune program reflecting organized antigen presentation, B cell maturation, and coordinated T cell effector activity.

Collectively, these findings establish that the maturation stage of TLS is paramount, with the GC-like TLS signature emerging as a robust, non-redundant biomarker identifying a patient subset with both a favorable prognosis and a significantly enhanced likelihood of immunotherapy benefit.

**Table 1:** Characteristic marker genes associated with different TLS maturation subtypes.

TLS Subtype	Dominant Cell Population	Functional Signature	Representative Marker Genes
GC-like TLS	Germinal center B cells, Tfh cells	Active B-cell proliferation, affinity maturation, class-switch recombination	CD19, BCL6, AICDA, MKI67, CXCL13, CD40LG, IL21, CXCR5, FCER2 (CD23), CR2 (CD21)
Primary follicle-like TLS	Naïve/mature B cells	B-cell aggregation without germinal center formation	MS4A1 (CD20), CD79A, CXCL13, CR2 (CD21), LTB
Early TLS	T cells, dendritic cells	Lymphocyte recruitment and early immune organization	CXCR5, CCL19, CCL21, CD3E, CD4, CD8A, CCR7

Abbreviations: TLS, tertiary lymphoid structure; GC, germinal center; Tfh, T follicular helper cells.

### 3.2 Local Independent Validation of Mature TLS Density as a Prognostic Marker in a Neoadjuvant PD-L1–Treated Cohort

Building upon the significant associations identified in IMvigor210 cohorts, we sought to prospectively validate the clinical relevance of mature TLSs in a well-annotated, real-world cohort of muscle-invasive bladder cancer (MIBC) patients from our institution who received neoadjuvant anti-PD-L1 therapy. This validation step was critical to assess the translational potential of TLS as a histopathological biomarker in a controlled clinical setting (Supplementary Table S1) [12,37].

We performed systematic histopathological evaluation of pretreatment tumor resection specimens (n = 60) using a standardized immunohistochemistry (IHC) panel (CD20/CD3/CD21/CD23/KI67) coupled with hematoxylin and eosin (H&E) staining. Mature TLSs were rigorously defined by the co-localization of organized B cell (CD20^+^) and T cell (CD3^+^) clusters with a distinct follicular dendritic cell network (CD21^+^). TLS density was quantified as the number of mature TLSs per square millimeter of viable tumor tissue and patients were stratified into high- and low-density groups based on the cohort median. In addition to the Follicular Dendritic Cell (FDC) network, Ki67-based assessment of proliferative centers provided an orthogonal readout supporting germinal-center activity, thereby anchoring “mature TLS” to functional architecture rather than morphology alone [36,38].

**Figure 2:**
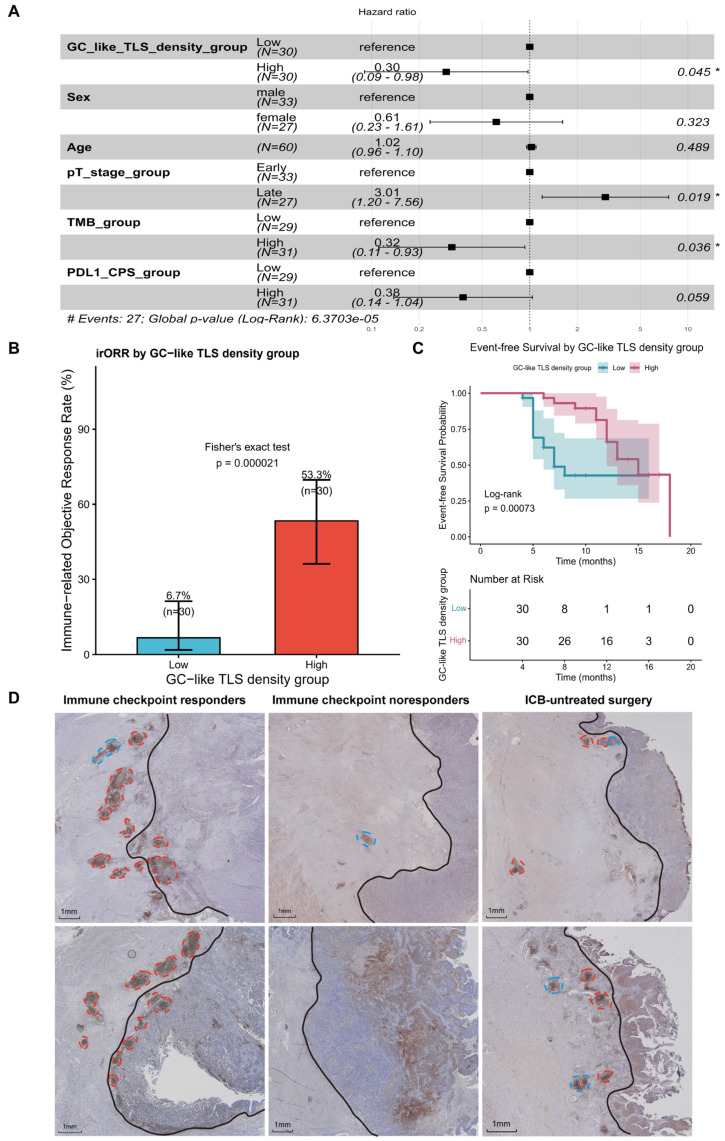
**Association Analysis of GC-like TLS Density with Clinical Outcomes and Hazard Ratios of Related Variables in Patients.** (**A**) Multivariate Cox regression forest plot (event-free survival): Squares = HR, horizontal lines = 95% CIs, vertical dashed line = HR = 1. *p < 0.05, (statistical significance). The proportional hazards (PH) assumption of Cox regression models was verified using Schoenfeld residuals, and no significant violations were detected. (**B**) Immune-related objective response rate (irORR) stratified by GC-like TLS density. *p* = 0.000021. (**C**) Kaplan–Meier curves of overall survival stratified by GC-like TLS density (95% CIs: shaded areas). *p* = 0.00073. (**D**) Schematic of TLS quantity, maturity and location in ICB-treated vs. ICB-untreated surgery patients: mature TLS (red circles); immature TLS (blue circles); tumor/non-tumor boundary (black curve) (Scale bar = 1 mm).

To ascertain the independent prognostic value of mature TLS density, we constructed a multivariate Cox proportional hazards model incorporating established clinical variables such as gender, age, clinical T stage, TMB, and baseline PD-L1 combined positive score (CPS) (Fig. 2A). In this model, high mature TLS density remained a powerful and independent predictor of favorable event-free survival (EFS) (Hazard Ratio [HR] = 0.30, 95% CI: 0.09–0.98, *p* = 0.045), outperforming PD-L1 CPS in this cohort. The clinical benefit was further reflected in the pathological response assessment, where the high TLS density group demonstrated a immune-related objective response rate (irORR) compared to the low-density group (53.3% vs. 6.7%, *p* < 0.001) (Fig. 2B). Consistent with the bioinformatics predictions, patients with high mature TLS density exhibited significantly prolonged event-free survival following neoadjuvant immunotherapy (Log-rank *p* < 0.001) (Fig. 2C). Consistent with the clinical response data shown in Fig. 2C, IHC analysis revealed that patients with favorable immune checkpoint inhibitor (ICI) responses exhibited a higher density of mature tertiary lymphoid structures (TLSs). Mature TLSs (red) were preferentially enriched at the tumor–normal tissue boundary, whereas immature TLSs (blue) were more diffusely distributed (Fig. 2D). Notably, responders to ICI therapy demonstrated prominent accumulation of mature TLSs along the invasive margin, while non-responders were characterized by a predominance of immature TLSs with no apparent spatial enrichment. These observations suggest that both the presence and spatial organization of mature TLSs are associated with improved ICI response.

In conclusion, this rigorous pathological validation in a prospectively managed clinical cohort robustly confirms that mature TLS density is a strong, independent, and clinically actionable biomarker for predicting benefit from neoadjuvant PD-L1 blockade in MIBC. Together with Result 3.1, this bridge transcriptomic “maturity signatures” and implementable pathology, enabling a two-track biomarker strategy (RNA-based vs. slide-based) depending on clinical context.

### 3.3 Functional Hierarchy of TLS Maturation Defined by Histopathological Transcriptomic Integration and Germinal Center–Dependent Adaptive Immunity

**Figure 3:**
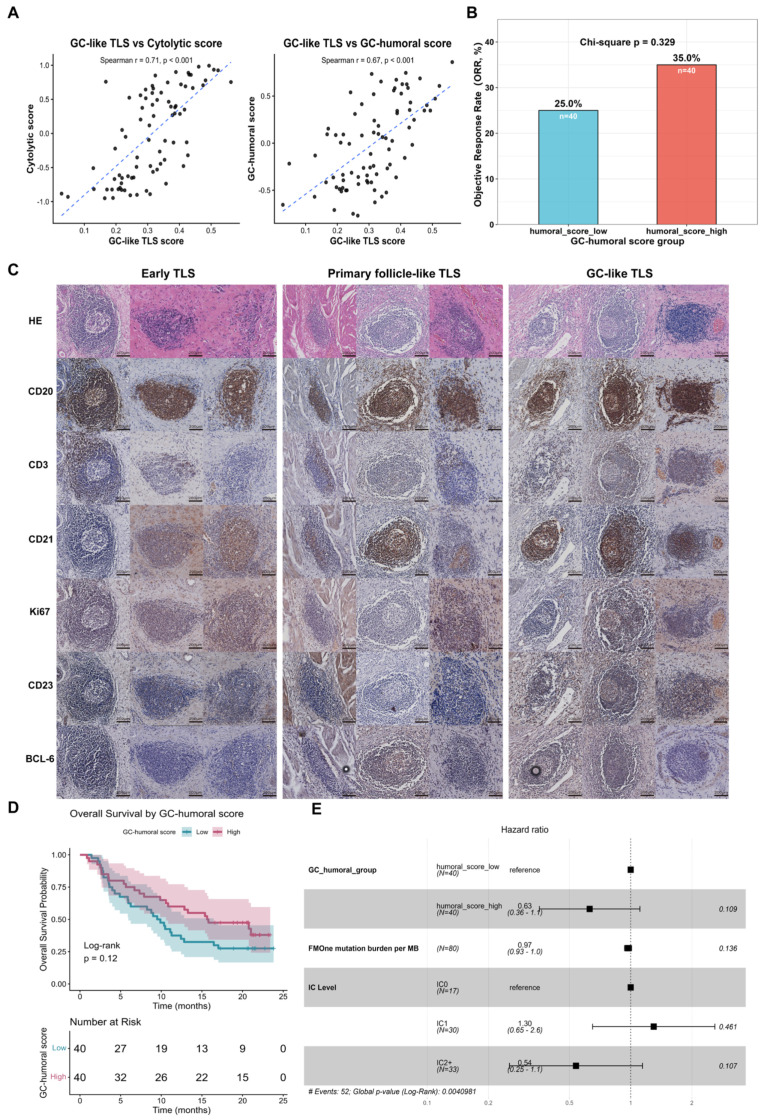
**Association Between GC-like TLS Score and Immune Function Scores, and the Impact of GC-humoral Score on Clinical Outcomes.** (**A**) Spearman correlations between GC-like TLS score and cytolytic score (left) and GC-humoral score (right). (**B**) Bar chart of objective response rate (ORR) across different GC-humoral score groups. (**C**) Histopathological Hematoxylin and Eosin staining (H&E) and immunohistochemical (IHC) staining images of different TLS architectures (Scale bar = 200 μm). (**D**) Kaplan–Meier overall-survival curves for GC-humoral low vs. high groups (95% CIs shaded). (**E**) Multivariate Cox regression forest plot (overall survival): Squares = HR point estimates, horizontal lines = 95% CIs, vertical dashed line = HR = 1. GC: germinal center; TLS: Tertiary lymphoid structure; CD: Cluster of Differentiation; BCL-6: B-cell lymphoma 6.

To mechanistically link TLS microstructure with its purported immune function, we performed an integrative analysis correlating high-resolution histopathological phenotyping with bulk transcriptomic immune signatures (Fig. 3A). This approach allowed us to define a functional hierarchy of TLS maturity and dissect its specific contributions to the adaptive immune landscape [39,40]. First, we developed and applied two complementary transcriptomic scores: a GC-Humoral Score, reflecting gene expression programs associated with B cell activation and antibody affinity maturation, and a Cytolytic Activity Score, representing effector functions of cytotoxic T and NK cells. Interestingly, both the GC-Humoral Score and Cytolytic Activity Score were significantly correlated with the GC-like TLS score. And the patients with high GC-Humoral Score had significantly higher ORR compared to those with lower GC-Humoral Score (Fig. 3B). This directly supports a “two-arm” model in which GC-like TLSs simultaneously enable B cell maturation and amplify cytotoxic effector programs, rather than reflecting isolated humoral activity. Then, based on a comprehensive immunohistochemistry panel (H&E, CD20, CD3, CD21, CD23, Ki67) [41,42], we assessed the immune function of TLSs. We precisely categorized TLS into three distinct maturation stages. Only structures meeting stringent criteria—including dense, organized clusters of B cells (CD20^+^) and T cells (CD3^+^), a well-defined network of follicular dendritic cells (FDCs) positive for both CD21 and CD23, and a central proliferative zone with high Ki67 expression—were classified as bona fide germinal center-like TLS (GC-like TLS) (Fig. 3C). This histopathological definition confirmed that GC-like TLSs represent the most advanced and functionally organized stage of TLS maturation within the tumor microenvironment.

Analysis demonstrated that in the immunotherapy cohort, a high GC-humoral score alone was not a statistically significant predictor of overall survival (Log-rank test, *p* = 0.12) (Fig. 3D), nor a significant discriminator of the objective response rate (Chi-square test, *p* = 0.329) (Fig. 3B); furthermore, it did not serve as an independent protective factor for overall survival (HR = 0.63, *p* = 0.109) (Fig. 3E). However, the mature TLS score exhibited a strong positive correlation with both this GC-humoral score (Spearman’s ρ = 0.67, *p* < 0.001) and the Cytolytic Activity Score (Spearman’s ρ = 0.71, *p* < 0.001) (Fig. 3A). It indicates that the presence of a structured GC, the anatomic substrate for the GC-Humoral Score, is almost invariably coupled with heightened cytotoxic effector function in the surrounding microenvironment. Conceptually, we interpret GC formation as the functional “switch” that couples antigen-focused B cell responses with efficient T cell priming and effector deployment [43]. The FDC network and activated B cells within the GC are posited to contribute to optimal antigen presentation and provision of co-stimulatory signals, thereby fostering the expansion and functional differentiation of tumor-antigen-specific cytotoxic T lymphocytes. This framework is also consistent with the increasing recognition that TLS ecosystems often include T follicular helper-like states and antigen-presenting B cells that jointly support durable cytotoxic immunity [44]. In conclusion, our integrated analysis establishes that TLS function is contingent upon its architectural maturity. The formation of a GC is a critical inflection point, transforming a TLS from a site of lymphocyte accumulation into a specialized synergistic activation center capable of orchestrating concurrent humoral and cell-mediated anti-tumor immunity. This mechanistic insight explains why the GC-like TLS phenotype, but not generalized lymphoid infiltration, serves as a powerful biomarker for productive anti-tumor immunity.

### 3.4 Pro-Inflammatory, Memory-Prone Immune Microenvironment Associated with GC-Like TLS and Reduced Local Immunosuppression

The presence of mature tertiary lymphoid structures is not merely a static histological feature but actively reprograms the broader tumor immune landscape. To delineate the systemic immunomodulatory impact of GC-like TLS, we performed a comprehensive deconvolution of bulk RNA-sequencing data combined with single-cell RNA-seq validation, revealing a profound and coordinated shift in the cellular composition and functional orientation of the tumor microenvironment (TME) [45,46].

**Figure 4:**
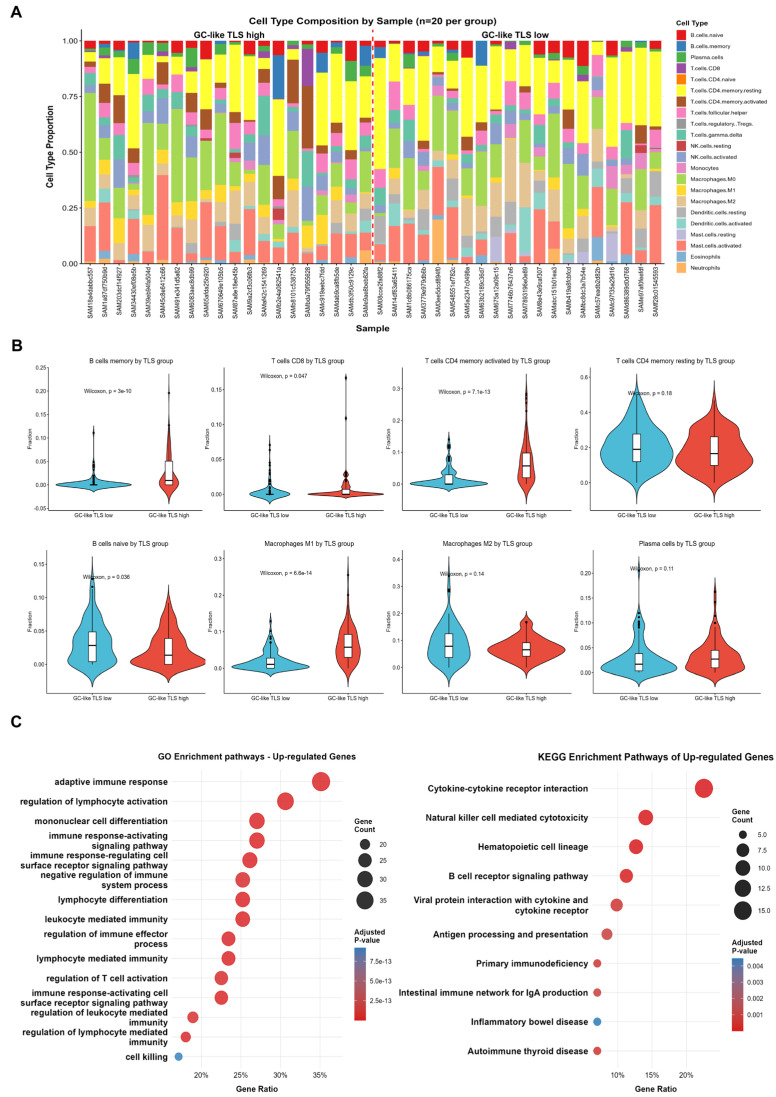
**Immune Cell Composition and Functional Enrichment Analysis of Upregulated Genes Across GC-like TLS Groups.** (**A**) Stacked bar plot showing sample-level cell-type composition inferred by CIBERSORT (LM22) for GC-like TLS high and low groups. (**B**) Violin plots showing immune cell proportions in GC-like TLS low and high groups. (**C**) Bubble plots of GO functional (left) and KEGG pathway (right) enrichment analyses for up-regulated genes. GC: Germinal center; TLS: Tertiary lymphoid structures; CD: Cluster of differentiation; CIBERSORT (LM22): LM22 leukocyte gene signature matrix; GO: Gene Ontology; KEGG: Kyoto Encyclopedia of Genes and Genomes.

A comparative analysis of leukocyte infiltration between tumors with high and low GC-like TLS signatures demonstrated a significant enrichment of lymphocyte subsets central to durable anti-tumor immunity (Fig. 4A). Most notably, we observed a marked increase in memory B cells (*p* < 0.001) and activated CD4^+^ memory T cells (*p* < 0.001) (Fig. 4B). To further characterize the functional state of the TME at single-cell resolution, we quantified the activity of Msigdb’s **HALLMARK_INFLAMMATORY_RESPONSE** pathway—a core signature of pro-inflammatory immune activation—across GC-like TLS high and low subgroups, and consistently detected significantly higher pathway activity in the GC-like TLS high subgroup (Supplementary Fig. S1, *p* < 0.001). This finding corroborates that GC-like TLS does not simply drive leukocyte accumulation, but rather elicits a functionally activated pro-inflammatory TME. The co-enrichment of these antigen-experienced, long-lived populations suggests that the GC-like TLS fosters a microenvironment conducive to immunological memory formation, which is critical for sustained tumor control and protection against recurrence. These findings provide a TIME-level explanation for the survival and response advantages observed in Results 3.1–3.2: GC-like TLSs are linked to “memory-capable” immunity rather than transient activation alone.

Beyond the adaptive arm, the innate immune compartment exhibited a significant phenotypic recalibration. Specifically, the proportion of pro-inflammatory M1-like macrophages was substantially elevated in GC-like TLS-high tumors (*p* < 0.001) (Fig. 4B). In contrast, the abundance of pro-tumorigenic M2-like macrophages remained unchanged, indicating a selective promotion of an anti-tumor myeloid phenotype rather than a blanket reduction in all macrophages. This shift is functionally critical, as M1 macrophages contribute to effector T cell recruitment, direct tumoricidal activity, and enhanced antigen presentation.

GO/KEGG enrichment analysis of up-regulated genes provided a mechanistic corroboration of these cellular findings (Fig. 4C). Tumors harboring mature TLS displayed significant upregulation of gene programs involved in adaptive immune response, lymphocyte activation and differentiation, B cell receptor signaling, and antigen processing and presentation via Major Histocompatibility Complex (MHC) class II. This transcriptomic signature confirms that the TLS-associated cellular shifts are underpinned by the activation of coherent, pro-inflammatory biological processes.

In summary, the mature GC-like TLS functions as an epicenter for immune microenvironmental reprogramming. It facilitates the recruitment and functional specialization of key adaptive immune cells toward memory and effector phenotypes while simultaneously instructing resident myeloid cells to adopt an anti-tumor, pro-inflammatory identity. This dual-action mechanism collectively engineers a highly immunocompetent TME that is primed for effective tumor cell recognition and elimination, thereby providing a cellular rationale for the superior clinical outcomes associated with TLS presence.

### 3.5 Chemokine-Mediated Cellular Crosstalk in TLS Maturation Defined by Integrated Transcriptomic and Spatial Analysis

The maturation of tertiary lymphoid structures (TLS) within the tumor microenvironment is regulated by the chemokine signaling axes CCL21–CCR7 and CXCL12–CXCR4 [47]. To elucidate the molecular mechanisms and cellular interactions driving TLS formation and maturation, we performed an integrated multi-omics analysis combining bulk RNA sequencing and single-cell RNA sequencing (scRNA-seq) data.

**Figure 5:**
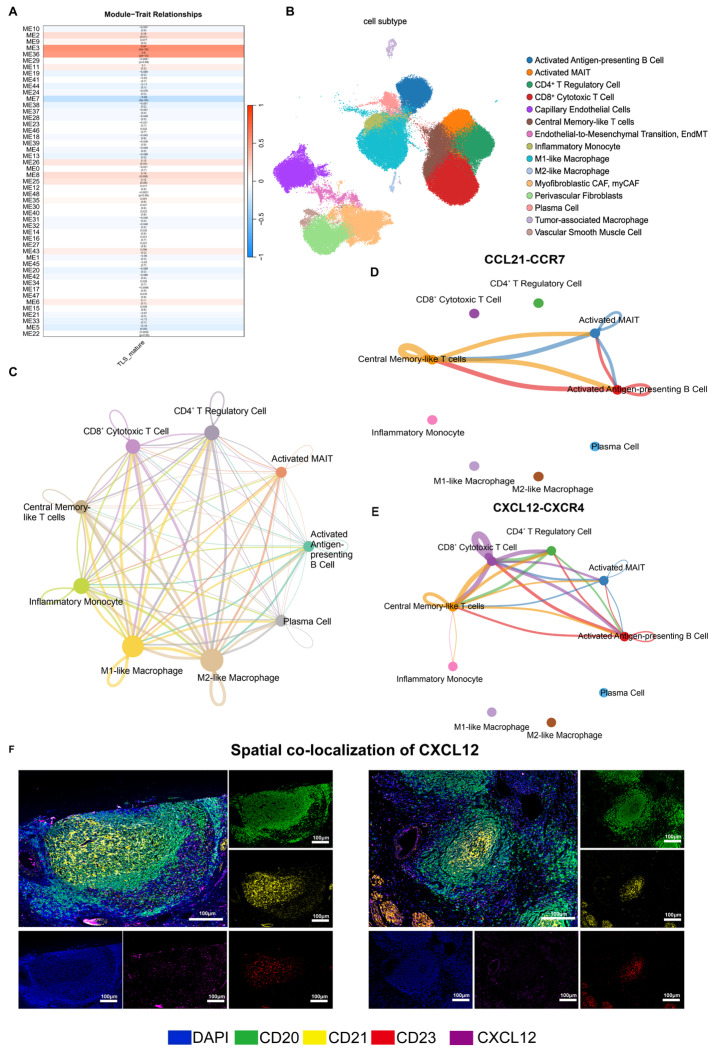
**Analysis of Cell Subtypes, Intercellular Communication, and Gene Co-expression Networks in the Tumor Microenvironment (TME).** (**A**) Heatmap of correlations between WGCNA module eigengenes (MEs) and the mature TLS phenotype. (**B**) TME cell subtype identification: UMAP plot with detailed annotations of stromal and immune cells (public scRNA-seq data). (**C**) CellChat analysis of intercellular communication networks (intersection gene set score > 0). Edge thickness = number of signaling pathways. (**D**) CCL21-CCR7 signaling axis: Expression and ligand-receptor communication across cell subtypes. (**E**) CXCL12-CXCR4 signaling axis: Ligand-receptor mediated intercellular communication. (**F**) Multiplex immunofluorescence staining of CXCL12 and immune cell markers in human bladder cancer tissues: merged image (upper left) surrounding single-channel images (Scale bar = 100 μm). TME: Tumor microenvironment; WGCNA: Weighted gene co-expression network analysis; TLS: Tertiary lymphoid structures; UMAP: Uniform Manifold Approximation and Projection; CCL21-CCR7: C-C motif chemokine ligand 21—C-C chemokine receptor 7 axis; CXCL12-CXCR4:C-X-C motif chemokine ligand 12—C-X-C chemokine receptor 4 axis.

First, weighted gene co-expression network analysis (WGCNA) was applied to identify gene modules associated with TLS status [48]. Two key modules, ME3 (r = 0.54, *p* < 0.001) and ME36 (r = 0.5, *p* < 0.001), showed significant positive correlation with a mature TLS phenotype (Fig. 5A). Functional enrichment analysis revealed that genes within these modules were highly enriched in immune-related pathways, particularly “chemokine signaling pathway”, “cytokine-cytokine receptor interaction”, and “B cell receptor signaling pathway” (Supplementary Fig. S2A), suggesting that these modules represent core transcriptional programs driving immune cell recruitment and organization.

To identify specific cellular subsets executing these transcriptional programs, we defined an “intersection gene set” based on the overlap between hub genes from ME3/ME36 and up-regulated genes in the mature TLS group. This gene set was then scored across cell subtypes identified in our scRNA-seq analysis (Fig. 5B). Activated antigen-presenting B cells, CD8^+^ cytotoxic T cells, and CD4^+^ regulatory T cells displayed the highest expression scores for TLS-associated genes (Fig. 5C, Supplementary Fig. S2B), indicating that these lymphocyte subsets are major contributors to the TLS-related gene modules.

CellChat analysis identified the CCL21–CCR7 and CXCL12–CXCR4 axes as the most prominent ligand–receptor pairs associated with these cellular clusters (Fig. 5D,E). Within the CCL21–CCR7 network, central memory-like T cells and activated MAIT cells acted as primary hubs, closely interacting with activated antigen-presenting B cells (Fig. 5D). Meanwhile, the CXCL12–CXCR4 network exhibited a broader connectivity pattern, with activated antigen-presenting B cells serving as key receptors receiving CXCL12 signals from diverse sources including CD8^+^ Cytotoxic T cell and CD4^+^ T Regulatory cell (Fig. 5E). These findings suggest a cooperative mechanism in which CCL21 and CXCL12 gradients guide the homing and spatial organization of B and T cells.

Finally, to spatially validate these mechanistic insights, we performed multiplex immunofluorescence (mIF) staining on tumor tissue sections. Imaging confirmed the presence of lymphocyte aggregates and revealed CXCL12 protein expression adjacent to CD20^+^ B cell clusters (Fig. 5F). This spatial co-localization corroborates our scRNA-seq-based inferences and confirms the *in situ* presence of CXCL12 in the microenvironment, supporting B cell aggregation and TLS assembly.

In addition to CCL21/CCL12 axes, recent literature highlights CXCL13 as a pivotal TLS-organizing chemokine and links CXCL13-associated TLS programs to immunotherapy responsiveness in bladder cancer, suggesting that multiple chemokine gradients may cooperate to stabilize the GC-like architecture [49]. We therefore propose a multi-axis maturation model in which CCL21–CCR7 supports lymphocyte recruitment and compartmentalization, CXCL12–CXCR4 sustains B cell clustering and GC-like organization, and CXCL13-related programs may reinforce follicular polarization and Tfh/B cell crosstalk [50], collectively driving the transition from nascent aggregates to GC-containing TLS.

In summary, our study integrates transcriptomic network and spatial analyses to demonstrate that the TLS maturation program is driven by specific chemokine signaling pathways. We propose that CCL21–CCR7 and CXCL12–CXCR4 pathways promote critical interactions between activated B cells and T cells, thereby regulating the structural organization of mature tertiary lymphoid structures in the tumor microenvironment.

### 3.6 LTβR Agonism–Induced TLS Maturation as a Strategy to Overcome Resistance to PD-L1 Blockade

**Figure 6:**
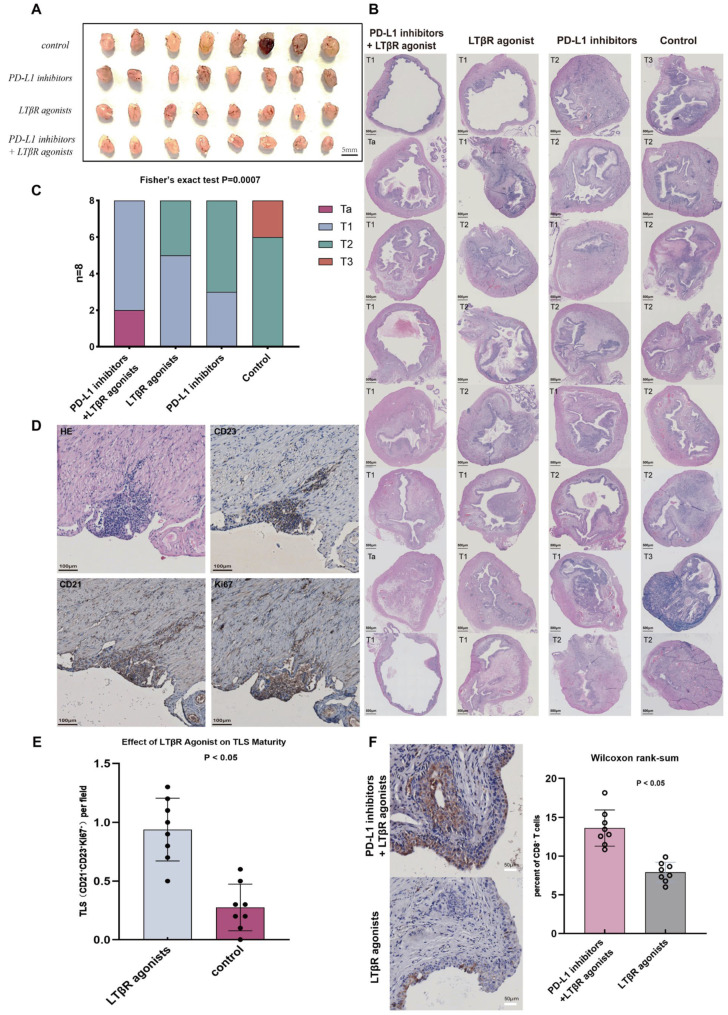
**Therapeutic effects of LTβR agonist ± PD-L1 inhibitor in tumor-bearing mice. (n = 8).** (**A**) Solid bladder images of tumor-bearing mice in different treatment groups. (**B**) Histopathological (H&E) staining images of tumor-bearing mice in different groups and their corresponding tumor stages (Scale bar: 500 μm). (**C**) Stacked bar chart showing the distribution of tumor stages among tumor-bearing mice in different treatment groups. (**D**) H&E (upper left) and immunohistochemical (IHC, CD23/CD21/Ki67) staining images of bladder sections from tumor-bearing mice in the LTβR agonist treatment group (Scale bar = 100 μm). (**E**) Bar chart showing the number of TLS (CD21^+^CD23^+^Ki67^+^) in IHC staining of tumor-bearing mice in the LTβR agonist treatment group and the control group (Wilcoxon test). (**F**) IHC staining images (left; CD8; Scale bar = 50 μm) of tumor-bearing mice in the LTβR agonist combined with PD-L1 inhibitor treatment group and the LTβR agonist treatment group, and bar graphs of the percentage of CD8^+^ T cells (right; Wilcoxon test). LTβR: lymphotoxin-βreceptor; PD-L1: Programmed Death-Ligand 1; H&E: Hematoxylin and Eosin; IHC: immunohistochemistry; Ki67: Ki-67 proliferation marker; CD: Cluster of Differentiation.

Based on the mechanistic insight that stromal-derived lymphotoxin signaling is central to TLS neogenesis, we hypothesized that pharmacologically activating this pathway could convert an immune-cold tumor microenvironment into one permissive for Immune Checkpoint Blockade (ICB) response [50,51]. We tested this therapeutic hypothesis in a syngeneic, immunocompetent murine model of bladder carcinoma characterized by low endogenous TLS infiltration and primary resistance to anti-PD-L1 monotherapy (Fig. 6A).

Mice bearing established tumors were randomized into four treatment arms: isotype control, PD-L1 inhibitor monotherapy, LTβR agonist monotherapy, and combination therapy with LTβR agonist plus PD-L1 inhibitor. As predicted, monotherapies showed limited efficacy, achieving only modest tumor growth delay. In stark contrast, the combination regimen induced profound and sustained tumor suppression. The superior anti-tumor efficacy of the combination was statistically significant in tumor progression (*p* < 0.001) (Fig. 6B,C).

Comprehensive histopathological and immunohistochemistry analysis of treated tumors elucidated the mechanism underlying this synergy. Tumors from the combination arm exhibited a significant shift towards mature, GC-like TLSs, evidenced by structured B/T cell zones, prominent FDC networks (CD21^+^/CD23^+^), and prolific Ki67^+^ proliferative centers (Fig. 6D,E). Quantification revealed that LTβR agonist treatment, either alone or in combination, significantly increased the total number of intratumoral TLSs per field as well as the proportion of CD8^+^ T cells compared with controls (Fig. 6F).

These data support a sequential, two-phase model of action. The LTβR agonist functions as a microenvironment-priming agent, stimulating the stromal compartment to initiate the transcriptional and secretory program necessary for structured lymphoid tissue formation. This creates a pre-conditioned, TLS-rich microenvironment characterized by enhanced antigen presentation and lymphocyte recruitment. Subsequently, the administration of PD-L1 blockade acts to release the brakes on the newly expanded and appropriately primed T cell populations, unleashing their full cytotoxic potential against tumor cells. The combination is thus greater than the sum of its parts, as neither agent alone can adequately provide both the necessary immunological infrastructure (TLS) and the effector cell liberation (checkpoint blockade).

In conclusion, this proof-of-concept study provides direct experimental evidence that targeting TLS biogenesis is a viable therapeutic strategy. The combination of an LTβR agonist with PD-L1 blockade demonstrates potent synergistic efficacy by fundamentally remodeling the immune tumor microenvironment from a state of ignorance/resistance to one of functional immune organization and responsiveness. These findings establish a strong preclinical rationale for translating “TLS-inducing therapy + ICB” combination regimens into clinical trials for bladder cancer and other ICB-resistant malignancies.

## 4 Discussion

Our study positions germinal center (GC)-like TLS maturity as both a predictive biomarker and a therapeutic lever in bladder cancer, linking immune architecture to clinical benefit across transcriptomic, pathological, and functional dimensions [52]. By emphasizing architectural maturity rather than TLS presence alone, we help reconcile the long-standing variability in TLS-associated clinical performance across tumor types and detection platforms: immature aggregates often reflect “infiltration,” whereas GC-like TLSs reflect “organized priming niches,” a distinction that is particularly consequential in bladder cancer where immune infiltration can coexist with suppressive myeloid programs and dysfunctional T cell states [53,54].

Recent advances in bladder cancer immunotherapy increasingly emphasize combination and biomarker-driven strategies. In addition to PD-(L)1 blockade, perioperative immunotherapy combinations and emerging immune targets beyond the PD-(L)1 axis are being actively explored, highlighting the need for robust biomarkers such as TLS maturation to guide patient stratification and therapeutic decision-making [55].

A major translational implication is biomarker stratification. In public cohorts, GC-like TLS transcriptional programs identified patients with superior survival and higher likelihood of response to PD-L1 blockade, and this concept translated cleanly into a pragmatic pathology metric in our neoadjuvant PD-L1 cohort, where mature TLS density independently predicted event-free survival and major pathological response, outperforming PD-L1 CPS [56]. This superiority extends across neoadjuvant settings of multiple cancers, addressing limitations of PD-L1/TMB and providing critical complementary value.

In resectable non-small cell lung cancer (NSCLC), Sun demonstrated that mature TLS independently predicts major pathological response (MPR), pathological complete response (pCR), and disease-free survival (DFS) [57]. In esophageal squamous cell carcinoma (ESCC), neoadjuvant chemoradiotherapy (NCRT) may affect the reliability of PD-L1 tumor proportion score (TPS) assessment due to treatment-induced alterations in tumor cellularity [58]. Huang validated that mature TLS (MTLS) alone outperforms both PD-L1 and TMB, as it reflects sustained antitumor immune activity post-treatment; notably, MTLS also complements a novel PD-L1 combined positive score (NCPS)—designed to capture PD-L1 on immune/stromal cells—resolving conventional PD-L1 scoring unreliability [59]. Extending these findings, Vanhersecke verified across 11 solid tumor types that mature TLS independently predicts immune checkpoint inhibitor (ICI) response, independent of PD-L1 or TMB, particularly in PD-L1-negative/low-TMB population [60].

Notably, recent literature advocates for integrating TLS into composite biomarker panels to boost predictive precision: Jiang showed the “High TLS + High TMB” subgroup in gastric cancer (GC) achieves optimal survival (AUC = 0.992) [61]. Similarly, Huang proposed an MTLS + NCPS panel for neoadjuvant ESCC, synergistically capturing immune activation (via MTLS) and adaptive resistance (via NCPS) [59]. Though TLS alone suffices for reliable prediction, its integration mitigates single-biomarker limitations, aligning with the trend toward multi-dimensional tumor immune landscape characterization.

Collectively, emerging evidence across tumor types suggests that mature/GC-like TLS represents a robust standalone biomarker in neoadjuvant settings and provides important complementary value to PD-L1 and TMB, particularly in biomarker-low or biomarker-negative populations.

Together, these findings support a two-track implementation strategy: slide-based GC-like TLS scoring using multiplex IHC criteria (B/T zoning, FDC networks, and Ki67^+^ proliferative centers) for routine pathology workflows, and RNA-based maturity signatures when tissue is limited or when centralized digital pathology is not feasible [12,14]. In practice, this enables flexible deployment across neoadjuvant, adjuvant, and metastatic settings, and offers a path to harmonize biomarker readouts between clinical trials and real-world care.

Recent advances in bladder cancer immunotherapy have rapidly expanded across disease stages [62]. In NMIBC, intravesical Bacillus Calmette–Guérin (BCG) remains the standard bladder-sparing immunotherapy, while multiple bladder-preservation options have emerged for BCG-unresponsive high-risk disease, including systemic PD-1 blockade (e.g., pembrolizumab) and intravesical gene-/cytokine-based immunotherapies (e.g., nadofaragene firadenovec and IL-15 agonist therapy in combination with BCG) [63]. In MIBC, perioperative immunotherapy is increasingly explored, with neoadjuvant checkpoint blockade (alone or with chemotherapy) and adjuvant PD-1 blockade (e.g., nivolumab after radical resection in high-risk urothelial carcinoma) representing key clinical directions [64]. In locally advanced/metastatic urothelial carcinoma, checkpoint inhibitors have become integrated into standard care through maintenance strategies after platinum-based chemotherapy (e.g., avelumab maintenance), and frontline combination regimens (e.g., enfortumab vedotin plus pembrolizumab) are reshaping first-line treatment paradigms [65]. Within this evolving landscape, our findings support a practical role for TLS maturation—particularly GC-like TLS—as an immune-architecture biomarker that can complement PD-L1/TMB and guide clinical decisions. Specifically, GC-like TLS maturity could be used for (i) baseline stratification to identify patients most likely to benefit from checkpoint blockade in both neoadjuvant and metastatic settings, (ii) selection of patients for combination strategies that aim to convert immune-cold tumors by inducing lymphoid organization (e.g., LTβR agonism plus PD-L1 blockade), and (iii) response monitoring/pharmacodynamic assessment, where on-treatment biopsies could evaluate maturation shifts toward GC-like TLS as an early indicator of effective immune remodeling [60]. This framework aligns with our proposed two-track implementation strategy using either pathology-based TLS maturity scoring or RNA-based maturity signatures depending on tissue availability and clinical workflow.

Importantly, the clinical context of our cohort aligns with the design of recent perioperative immunotherapy trials in bladder cancer, in which immune checkpoint blockade is administered as a first-line systemic therapy in the neoadjuvant setting [66]. Similar to contemporary studies, our population predominantly consisted of treatment-naïve MIBC patients, with limited prior systemic therapy and preserved performance status. However, a proportion of patients had prior exposure to intravesical BCG, reflecting real-world disease trajectories. These features suggest that our cohort is broadly representative of patients considered for perioperative immunotherapy, supporting the generalizability of our findings [67].

In parallel with advances in tissue-based immune architecture profiling, minimally invasive liquid biopsy approaches are increasingly shaping clinical practice in uro-oncology [68]. Urine- and blood-based assays, including circulating tumor DNA (ctDNA), cell-free DNA (cfDNA), and other tumor- or immune-derived analytes, provide dynamic and longitudinal measures of tumor burden, minimal residual disease, and treatment response. These platforms offer a systemic and time-resolved perspective that complements the spatially resolved but static information derived from tumor tissue [69]. Within this framework, GC-like TLS maturity may be interpreted as a structural indicator of the tumor’s capacity for local immune priming, whereas liquid biopsy readouts reflect ongoing tumor kinetics and therapy-induced changes. Integrating TLS maturity assessment with serial ctDNA or urine-based molecular measurements could therefore enable a multimodal monitoring strategy combining immune architectural state with noninvasive tumor burden tracking [60]. Such an approach may be particularly informative in neoadjuvant settings, where early molecular clearance signals could be contextualized by underlying immune organization, and in metastatic disease, where dynamic ctDNA changes may help distinguish true progression from immune-related pseudoprogression. Furthermore, in TLS-low or TLS-immature tumors, rising ctDNA or urine-based molecular signals during therapy might provide early indication of inadequate immune activation or emerging resistance to PD-(L)1 blockade [70]. Circulating tumor DNA (ctDNA), cell-free DNA (cfDNA), and circulating immune signatures enable non-invasive monitoring of tumor burden and treatment response. Integrating these dynamic biomarkers with tissue-derived GC-like TLS metrics could allow longitudinal tracking of immune activation and early detection of immune remodeling or resistance during immunotherapy, thereby improving biomarker-guided treatment strategies [71]. Prospective studies incorporating both tissue-defined TLS metrics and longitudinal liquid biopsy assays may thus refine response prediction, minimal residual disease detection, and treatment decision-making within a multimodal precision immunotherapy framework.

Our mechanistic observations sharpen how such a biomarker should be interpreted clinically. The strong coupling between GC-humoral programs and cytolytic activity suggests that GC-like TLSs operate as an integrated immune unit rather than a purely B cell phenomenon. This provides a mechanistic rationale for why TLS maturity improves prediction of ICI benefit: checkpoint release is more likely to act on appropriately primed, tumor-reactive T cell populations when local priming infrastructure is present. From a trial-design perspective, this argues that GC-like TLS maturity is not only a baseline predictor but may also serve as a pharmacodynamic marker for therapies intended to restructure the tumor immune microenvironment.

Our chemokine and communication analyses further inform therapeutic strategies. We identify CCL21–CCR7 and CXCL12–CXCR4 axes as prominent contributors to recruitment and spatial organization (Fig. 5D,E). These pathways are interpreted within a broader TLS maturation circuitry. In this framework, stromal organizer programs, lymphotoxin signaling, and follicular chemokines cooperate to stabilize follicular polarization, follicular dendritic cell (FDC) networks, and germinal center (GC) persistence. Importantly, emerging evidence suggests that post-transcriptional regulatory mechanisms, including microRNAs (miRNAs), may represent an additional layer controlling TLS development. Specific miRNAs have been implicated in regulating B-cell activation, T follicular helper (Tfh) cell differentiation, and chemokine expression within the tumor microenvironment. For example, miRNA-dependent modulation of chemokine signaling pathways such as CXCL13–CXCR5, CCL21–CCR7, and CXCL12–CXCR4 may influence lymphocyte recruitment and the stability of FDC networks, thereby shaping germinal center–like niches within TLSs. In this framework, miRNA-regulated chemokine signaling could cooperate with lymphotoxin-driven stromal programs to coordinate B–T cell crosstalk and sustain GC-like TLS maturation. These multilayer regulatory circuits further support the concept that TLS formation reflects an organized immune ecosystem rather than a simple accumulation of lymphoid cells [72].

Therapeutically, our LTβR agonism experiments provide proof-of-concept that TLS maturation is inducible and can overcome resistance to PD-L1 blockade [73] (Fig. 6D,E). These results support an “infrastructure-plus-release” paradigm, in which LTβR-driven microenvironmental priming establishes TLS-rich, antigen-presenting architecture. Checkpoint blockade is then applied to unleash cytotoxic effector function. This framework is directly actionable for clinical development. It supports combination regimens that include TLS-inducing agents. It also suggests that on-treatment biopsies should assess more than immune-cell abundance. TLS maturity and shifts toward GC-like states should be evaluated as early indicators of biological engagement.

Several practical considerations follow for clinical translation. First, TLS evaluation requires standardization across centers. A feasible path is a tiered scoring system capturing density and maturity (and, where possible, spatial context), with harmonized staining panels and digital quantification to reduce inter-observer variability.

Second, B cells can exhibit both antitumor and regulatory phenotypes. Therefore, biomarker refinement should include B-cell state composition within TLSs. Relevant features include APC-like B cells, plasma cell differentiation, and potential regulatory programs.

Third, trial frameworks should prospectively test whether induced TLSs achieve GC competence rather than merely increasing lymphoid aggregates; this demands longitudinal sampling and pharmacodynamic markers that track FDC network formation and proliferative GC activity.

In summary, our study supports a paradigm shift in how the tumor immune microenvironment is understood and targeted in bladder cancer immunotherapy, from a quantitative census of infiltrating leukocytes toward a spatially organized and therapeutically malleable immune architecture. Through integrated multi-cohort transcriptomics, histopathology-driven TLS phenotyping, single-cell mapping, and *in vivo* perturbation, we establish that GC-like TLS maturity constitutes a functional nexus where localized humoral immunity converges with cytotoxic effector programs. This coordinated activity establishes an *in situ* priming infrastructure that maximizes susceptibility to immune checkpoint blockade. Thus, GC-like TLS maturity represents not only a robust prognostic and predictive biomarker, but more importantly, a therapeutically engineerable immune state, opening a direct path to converting immune-cold tumors into checkpoint-responsive ecosystems.

Practically, our data support an “infrastructure-plus-release” paradigm. In this framework, TLS-inducing strategies first establish GC-competent lymphoid organization. PD-(L)1 blockade then consolidates this architecture to elicit durable tumor control. Looking ahead, a key translational priority will be to standardize maturity-aware TLS scoring across centers. Longitudinal sampling should be incorporated to capture on-treatment maturation dynamics as a pharmacodynamic readout. Prospective trials will be required to determine whether converting “TLS-low/immature” tumors into “GC-like TLS-high” tumors translates into improved response and survival.

Several limitations warrant consideration. First, portions of the transcriptomic and clinical datasets were analyzed retrospectively, which may introduce selection bias and limit causal inference. Second, although our findings support the predictive value of TLS maturity, prospective validation across diverse immunotherapy regimens—including combination and neoadjuvant settings—remains necessary. Third, robust and validated non-invasive correlates of TLS maturation are currently lacking, constraining real-time monitoring in clinical practice.

These limitations define clear directions for future research. Prospective clinical studies should integrate GC-like TLS assessment with contemporary immunotherapy strategies and serial sampling designs. Parallel efforts to develop liquid-biopsy–based surrogates of TLS maturation—such as circulating immune signatures or B-cell–related markers—may enable dynamic, non-invasive tracking of immune-architectural remodeling during therapy. Collectively, these steps will help determine whether therapeutically engineering TLS maturation can be translated into broader and more durable benefit from checkpoint immunotherapy across both resectable and metastatic disease.

## 5 Conclusion

In conclusion, our integrative multi-cohort analyses establish that germinal center (GC)-like TLS maturity, rather than TLS presence alone, represents a functionally meaningful immune-architectural state that is tightly linked to improved survival and heightened responsiveness to PD-L1 blockade in bladder cancer. In a real-world neoadjuvant PD-L1–treated MIBC cohort, pathology-defined mature TLS density independently predicted major pathological response and event-free survival, outperforming PD-L1 CPS and providing a clinically implementable biomarker readout. Mechanistically, GC-like TLS formation marks a functional transition in which humoral programs become coupled to cytotoxic effector activity and a pro-inflammatory, memory-prone tumor immune microenvironment, supported by chemokine-mediated cellular organization involving the CCL21–CCR7 and CXCL12–CXCR4 axes. Importantly, therapeutic activation of LTβR signaling promoted TLS maturation and sensitized otherwise resistant tumors to PD-L1 blockade *in vivo*, supporting an actionable “infrastructure-plus-release” strategy in which TLS-inducing priming is consolidated by checkpoint inhibition. Together, these findings position GC-like TLS maturity as both a measurable biomarker and a druggable axis for precision immunotherapy in bladder cancer, providing a rational framework for future prospective trials that standardize maturity-aware TLS scoring and test TLS-inducing combinations to broaden checkpoint benefit across disease stages.

## Data Availability

The datasets generated and/or analyzed during the current study are available from the corresponding author on reasonable request.

## Supplementary Materials

The supplementary material is available online at https://www.techscience.com/doi/10.32604/or.2026.077808/s1.

## References

[1] Yi M , Zheng X , Niu M , Zhu S , Ge H , Wu K . Combination strategies with PD-1/PD-L1 blockade: Current advances and future directions. Mol Cancer. 2022; 21( 1): 28. doi:10.1186/s12943-021-01489-2. 35062949 PMC8780712

[2] Grayson M . Bladder cancer. Nature. 2017; 551( 7679): S33. doi:10.1038/551S33a. 29117156

[3] Cho SF , Anderson KC , Tai YT . Microenvironment is a key determinant of immune checkpoint inhibitor response. Clin Cancer Res. 2022; 28( 8): 1479– 81. doi:10.1158/1078-0432.CCR-22-0015. 35121621

[4] Petroni G , Pillozzi S , Antonuzzo L . Exploiting tertiary lymphoid structures to stimulate antitumor immunity and improve immunotherapy efficacy. Cancer Res. 2024; 84( 8): 1199– 209. doi:10.1158/0008-5472.CAN-23-3325. 38381540 PMC11016894

[5] Lin J , Jiang S , Chen B , Du Y , Qin C , Song Y , et al. Tertiary lymphoid structures are linked to enhanced antitumor immunity and better prognosis in muscle-invasive bladder cancer. Adv Sci. 2025; 12( 7): 2410998. doi:10.1002/advs.202410998. PMC11831474 39739621

[6] Ghajar-Rahimi G , Patel I , Yusuf N . Tertiary lymphoid structures in human melanoma: Molecular mechanisms and therapeutic opportunities. Cells. 2025; 14( 17): 1378. doi:10.3390/cells14171378. 40940789 PMC12428040

[7] Teng X , Chen Z , Bai Y , Cao H , Zhang J , Xu L , et al. Tertiary lymphoid structures as independent predictors of favorable prognosis in muscle-invasive bladder cancer. Cancer Med. 2025; 14( 10): e70978. doi:10.1002/cam4.70978. 40396416 PMC12093152

[8] Shu DH , Sidiropoulos DN . Maturation of tertiary lymphoid structures. Methods Mol Biol. 2025; 2864: 43– 55. doi:10.1007/978-1-0716-4184-2_3. 39527216

[9] Zhang Y , Liu G , Zeng Q , Wu W , Lei K , Zhang C , et al. CCL19-producing fibroblasts promote tertiary lymphoid structure formation enhancing anti-tumor IgG response in colorectal cancer liver metastasis. Cancer Cell. 2024; 42( 8): 1370– 85.e9. doi:10.1016/j.ccell.2024.07.006. 39137726

[10] Lin X , Kang K , Chen P , Zeng Z , Li G , Xiong W , et al. Regulatory mechanisms of PD-1/PD-L1 in cancers. Mol Cancer. 2024; 23( 1): 108. doi:10.1186/s12943-024-02023-w. 38762484 PMC11102195

[11] Jiang Y , Chen M , Nie H , Yuan Y . PD-1 and PD-L1 in cancer immunotherapy: Clinical implications and future considerations. Hum Vaccines Immunother. 2019; 15( 5): 1111– 22. doi:10.1080/21645515.2019.1571892. PMC6605868 30888929

[12] Rosenberg JE , Galsky MD , Powles T , Petrylak DP , Bellmunt J , Loriot Y , et al. Atezolizumab monotherapy for metastatic urothelial carcinoma: Final analysis from the phase II IMvigor210 trial. ESMO Open. 2024; 9( 12): 103972. doi:10.1016/j.esmoop.2024.103972. 39642637 PMC11667038

[13] Huang A , Sun Z , Hong H , Yang Y , Chen J , Gao Z , et al. Novel hypoxia- and lactate metabolism-related molecular subtyping and prognostic signature for colorectal cancer. J Transl Med. 2024; 22( 1): 587. doi:10.1186/s12967-024-05391-5. 38902737 PMC11191174

[14] Li X , Chu X , Xu W , Yang Y , Wei T , Bo Y , et al. Integrated spatial transcriptomic profiling to dissect the cellular characteristics of tumor-associated tertiary lymphoid structures. Cell Rep. 2025; 44( 9): 116250. doi:10.1016/j.celrep.2025.116250. 40934085

[15] Tang Z , Bai Y , Fang Q , Yuan Y , Zeng Q , Chen S , et al. Spatial transcriptomics reveals tryptophan metabolism restricting maturation of intratumoral tertiary lymphoid structures. Cancer Cell. 2025; 43( 6): 1025– 44.e14. doi:10.1016/j.ccell.2025.03.011. 40185093

[16] Deng S , Chen Y , Song B , Wang H , Huang S , Wu K , et al. Tertiary lymphoid structures in cancer: Spatiotemporal heterogeneity, immune orchestration, and translational opportunities. J Hematol Oncol. 2025; 18( 1): 97. doi:10.1186/s13045-025-01754-7. 41219991 PMC12606831

[17] Jager KJ , van Dijk PC , Zoccali C , Dekker FW . The analysis of survival data: The Kaplan–Meier method. Kidney Int. 2008; 74( 5): 560– 5. doi:10.1038/ki.2008.217. 18596735

[18] Yoshihara K , Shahmoradgoli M , Martínez E , Vegesna R , Kim H , Torres-Garcia W , et al. Inferring tumour purity and stromal and immune cell admixture from expression data. Nat Commun. 2013; 4: 2612. doi:10.1038/ncomms3612. 24113773 PMC3826632

[19] Hänzelmann S , Castelo R , Guinney J . GSVA gene set variation analysis for microarray and RNA-Seq data. BMC Bioinform. 2013; 14( 1): 7. doi:10.1186/1471-2105-14-7. PMC3618321 23323831

[20] Narayanan S , Kawaguchi T , Yan L , Peng X , Qi Q , Takabe K . Cytolytic activity score to assess anticancer immunity in colorectal cancer. Ann Surg Oncol. 2018; 25( 8): 2323– 31. doi:10.1245/s10434-018-6506-6. 29770915 PMC6237091

[21] Newman AM , Liu CL , Green MR , Gentles AJ , Feng W , Xu Y , et al. Robust enumeration of cell subsets from tissue expression profiles. Nat Methods. 2015; 12( 5): 453– 7. doi:10.1038/nmeth.3337. 25822800 PMC4739640

[22] Aran D , Hu Z , Butte AJ . xCell: Digitally portraying the tissue cellular heterogeneity landscape. Genome Biol. 2017; 18( 1): 220. doi:10.1186/s13059-017-1349-1. 29141660 PMC5688663

[23] Love MI , Huber W , Anders S . Moderated estimation of fold change and dispersion for RNA-seq data with DESeq2. Genome Biol. 2014; 15( 12): 550. doi:10.1186/s13059-014-0550-8. 25516281 PMC4302049

[24] Liu S , Wang Z , Zhu R , Wang F , Cheng Y , Liu Y . Three differential expression analysis methods for RNA sequencing: Limma, EdgeR, DESeq2. J Vis Exp. 2021. doi:10.3791/62528. 34605806

[25] Wu T , Hu E , Xu S , Chen M , Guo P , Dai Z , et al. clusterProfiler 4.0: A universal enrichment tool for interpreting omics data. Innovation. 2021; 2( 3): 100141. doi:10.1016/j.xinn.2021.100141. 34557778 PMC8454663

[26] Feng S , Xu Y , Dai Z , Yin H , Zhang K , Shen Y . Integrative analysis from multicenter studies identifies a WGCNA-derived cancer-associated fibroblast signature for ovarian cancer. Front Immunol. 2022; 13: 951582. doi:10.3389/fimmu.2022.951582. 35874760 PMC9304893

[27] Wolf FA , Angerer P , Theis FJ . SCANPY large-scale single-cell gene expression data analysis. Genome Biol. 2018; 19( 1): 15. doi:10.1186/s13059-017-1382-0. 29409532 PMC5802054

[28] Korsunsky I , Millard N , Fan J , Slowikowski K , Zhang F , Wei K , et al. Fast, sensitive and accurate integration of single-cell data with Harmony. Nat Methods. 2019; 16( 12): 1289– 96. doi:10.1038/s41592-019-0619-0. 31740819 PMC6884693

[29] Floudas A , Smith C , Tynan O , Neto N , Krishna V , Wade S , et al. Distinct stromal and immune cell interactions shape the pathogenesis of rheumatoid and psoriatic arthritis. Ann Rheum Dis. 2022; 81: 1224– 42. doi:10.1136/annrheumdis-2022-eular.1811. 35701153

[30] Yu X , Chen YA , Conejo-Garcia JR , Chung CH , Wang X . Estimation of immune cell content in tumor using single-cell RNA-seq reference data. BMC Cancer. 2019; 19( 1): 715. doi:10.1186/s12885-019-5927-3. 31324168 PMC6642583

[31] Jin S , Plikus MV , Nie Q . CellChat for systematic analysis of cell–cell communication from single-cell transcriptomics. Nat Protoc. 2025; 20( 1): 180– 219. doi:10.1038/s41596-024-01045-4. 39289562

[32] Fang Z , Tian Y , Sui C , Guo Y , Hu X , Lai Y , et al. Single-cell transcriptomics of proliferative phase endometrium: Systems analysis of cell–cell communication network using CellChat. Front Cell Dev Biol. 2022; 10: 919731. doi:10.3389/fcell.2022.919731. 35938159 PMC9352955

[33] Jin S , Guerrero-Juarez CF , Zhang L , Chang I , Ramos R , Kuan CH , et al. Inference and analysis of cell-cell communication using CellChat. Nat Commun. 2021; 12: 1088. doi:10.1038/s41467-021-21246-9. 33597522 PMC7889871

[34] Napolitano S , Matrone N , Muddassir AL , Martini G , Sorokin A , De Falco V , et al. Triple blockade of EGFR, MEK and PD-L1 has antitumor activity in colorectal cancer models with constitutive activation of MAPK signaling and PD-L1 overexpression. J Exp Clin Cancer Res. 2019; 38( 1): 492. doi:10.1186/s13046-019-1497-0. 31842958 PMC6915948

[35] Deng L , Liang H , Burnette B , Beckett M , Darga T , Weichselbaum RR , et al. Irradiation and anti–PD-L1 treatment synergistically promote antitumor immunity in mice. J Clin Investig. 2014; 124( 2): 687– 95. doi:10.1172/JCI67313. 24382348 PMC3904601

[36] Ma G , Jia H , Zhang G , Liang Y , Dong X , Fu G , et al. Presence, subtypes, and prognostic significance of tertiary lymphoid structures in urothelial carcinoma of the bladder. Oncologist. 2024; 29( 2): e248– 58. doi:10.1093/oncolo/oyad283. 37874923 PMC10836299

[37] Sotelo M , Alonso-Gordoa T , Gajate P , Gallardo E , Morales-Barrera R , Pérez-Gracia JL , et al. Atezolizumab in locally advanced or metastatic urothelial cancer: A pooled analysis from the Spanish patients of the IMvigor 210 cohort 2 and 211 studies. Clin Transl Oncol. 2021; 23( 4): 882– 91. doi:10.1007/s12094-020-02482-9. 32897497 PMC7979625

[38] Dai S , Zeng H , Liu Z , Jin K , Jiang W , Wang Z , et al. Intratumoral CXCL13^+^CD8^+^T cell infiltration determines poor clinical outcomes and immunoevasive contexture in patients with clear cell renal cell carcinoma. J Immunother Cancer. 2021; 9( 2): e001823. doi:10.1136/jitc-2020-001823. 33589528 PMC7887366

[39] van Dijk N , Gil-Jimenez A , Silina K , van Montfoort ML , Einerhand S , Jonkman L , et al. The tumor immune landscape and architecture of tertiary lymphoid structures in urothelial cancer. Front Immunol. 2021; 12: 793964. doi:10.3389/fimmu.2021.793964. 34987518 PMC8721669

[40] Fridman WH , Meylan M , Petitprez F , Sun CM , Italiano A , Sautès-Fridman C . B cells and tertiary lymphoid structures as determinants of tumour immune contexture and clinical outcome. Nat Rev Clin Oncol. 2022; 19( 7): 441– 57. doi:10.1038/s41571-022-00619-z. 35365796

[41] Yüceer RO , Başpınar Ş . Investigation of Ki67 and phospho-histone H3 expressions in urothelial carcinoma of the bladder by immunohistochemical method. Cureus. 2024; 16( 2): e55297. doi:10.7759/cureus.55297. 38558732 PMC10981782

[42] Ziaran S , Harsanyi S , Bevizova K , Varchulova Novakova Z , Trebaticky B , Bujdak P , et al. Expression of E-cadherin, Ki-67, and p53 in urinary bladder cancer in relation to progression, survival, and recurrence. Eur J Histochem. 2020; 64( 2): 3098. doi:10.4081/ejh.2020.3098. 32214283 PMC7118433

[43] Yan X , Zhang X , Wu HH , Wu SJ , Tang XY , Liu TZ , et al. Novel T-cell signature based on cell pair algorithm predicts survival and immunotherapy response for patients with bladder urothelial carcinoma. Front Immunol. 2022; 13: 994594. doi:10.3389/fimmu.2022.994594. 36466869 PMC9712189

[44] Cui C , Craft J , Joshi NS . T follicular helper cells in cancer, tertiary lymphoid structures, and beyond. Semin Immunol. 2023; 69: 101797. doi:10.1016/j.smim.2023.101797. 37343412

[45] Teillaud JL , Houel A , Panouillot M , Riffard C , Dieu-Nosjean MC . Tertiary lymphoid structures in anticancer immunity. Nat Rev Cancer. 2024; 24( 9): 629– 46. doi:10.1038/s41568-024-00728-0. 39117919

[46] Zhang L , Ren S , Lan T , Marco V , Liu N , Wei B , et al. Mature tertiary lymphoid structures linked to HPV status and anti-PD-1 based chemoimmunotherapy response in head and neck squamous cell carcinoma. OncoImmunology. 2025; 14: 2528109. doi:10.1080/2162402X.2025.2528109. 40621740 PMC12239792

[47] Zhao L , Qiu Z , Yang Z , Xu L , Pearce TM , Wu Q , et al. Lymphatic endothelial-like cells promote glioblastoma stem cell growth through cytokine-driven cholesterol metabolism. Nat Cancer. 2024; 5( 1): 147– 66. doi:10.1038/s43018-023-00658-0. 38172338

[48] Langfelder P , Horvath S . WGCNA: An R package for weighted correlation network analysis. BMC Bioinform. 2008; 9( 1): 559. doi:10.1186/1471-2105-9-559. PMC2631488 19114008

[49] Gao SH , Liu SZ , Wang GZ , Zhou GB . CXCL13 in cancer and other diseases: Biological functions, clinical significance, and therapeutic opportunities. Life. 2021; 11( 12): 1282. doi:10.3390/life11121282. 34947813 PMC8708574

[50] Zuo M , Wang AA , Gommerman JL . Follicle on the roof: Tertiary lymphoid structures in central nervous system autoimmunity. Immunol Rev. 2025; 332: e70045. doi:10.1111/imr.70045. 40568975 PMC12199550

[51] Nayak S , Calvo JA , Cantor SB . Targeting translesion synthesis (TLS) to expose replication gaps, a unique cancer vulnerability. Expert Opin Ther Targets. 2021; 25( 1): 27– 36. doi:10.1080/14728222.2021.1864321. 33416413 PMC7837368

[52] Zhang L , Zhang R , Jin D , Zhang T , Shahatiaili A , Zang J , et al. Synergistic induction of tertiary lymphoid structures by chemoimmunotherapy in bladder cancer. Br J Cancer. 2024; 130( 7): 1221– 31. doi:10.1038/s41416-024-02598-7. 38332180 PMC10991273

[53] Klein C , Mebroukine S , Madéry M , Moisand A , Boyer T , Larmonier N , et al. Myeloid-derived suppressor cells in bladder cancer: An emerging target. Cells. 2024; 13( 21): 1779. doi:10.3390/cells13211779. 39513886 PMC11544784

[54] Liu J , Shi Z , Li Y , Ma J , Yao J , Yuan Z , et al. High-resolution transcriptome atlas of bladder cancer highlights the functional myeloid subsets in modulating immune microenvironment. eBioMedicine. 2025; 117: 105801. doi:10.1016/j.ebiom.2025.105801. 40561775 PMC12240107

[55] Ghaneialvar H , Jahani S , Hashemi E , Khalilzad MA , Falahi S , Rashidi MA , et al. Combining anti-checkpoint immunotherapies and cancer vaccines as a novel strategy in oncological therapy: A review. Hum Immunol. 2025; 86( 1): 111209. doi:10.1016/j.humimm.2024.111209. 39662393

[56] Smith ME , Farahani SJ , Chao T , Palmer M , Arriola A , Lal P . PD-L1 positivity associated with presence of tertiary lymphoid structures and high-stage disease in upper tract urothelial carcinoma. Am J Clin Pathol. 2020; 154( 6): 802– 10. doi:10.1093/ajcp/aqaa105. 32864684

[57] Sun X , Liu W , Sun L , Mo H , Feng Y , Wu X , et al. Maturation and abundance of tertiary lymphoid structures are associated with the efficacy of neoadjuvant chemoimmunotherapy in resectable non-small cell lung cancer. J Immunother Cancer. 2022; 10( 11): e005531. doi:10.1136/jitc-2022-005531. 37011953 PMC9644367

[58] Huang WT , Lu HI , Wang YM , Chen YH , Lo CM , Lin WC , et al. Positive programmed cell death-ligand 1 expression predicts poor treatment outcomes in esophageal squamous cell carcinoma patients receiving neoadjuvant chemoradiotherapy. J Clin Med. 2019; 8( 11): 1864. doi:10.3390/jcm8111864. 31684197 PMC6912507

[59] Huang H , Zhao G , Wang T , You Y , Zhang T , Chen X , et al. Survival benefit and spatial properties of tertiary lymphoid structures in esophageal squamous cell carcinoma with neoadjuvant therapies. Cancer Lett. 2024; 601: 217178. doi:10.1016/j.canlet.2024.217178. 39142497

[60] Vanhersecke L , Brunet M , Guégan JP , Rey C , Bougouin A , Cousin S , et al. Mature tertiary lymphoid structures predict immune checkpoint inhibitor efficacy in solid tumors independently of PD-L1 expression. Nat Cancer. 2021; 2( 8): 794– 802. doi:10.1038/s43018-021-00232-6. 35118423 PMC8809887

[61] Jiang Q , Tian C , Wu H , Min L , Chen H , Chen L , et al. Tertiary lymphoid structure patterns predicted anti-PD1 therapeutic responses in gastric cancer. Chin J Cancer Res. 2022; 34( 3): 365– 82. doi:10.21147/j.issn.1000-9604.2022.04.05. 36199531 PMC9468020

[62] Meng L , Zhu X , Ji X , Wang B , Zhang H , Zhang G , et al. Advances in the immunological microenvironment and immunotherapy of bladder cancer. Front Immunol. 2025; 16: 1609871. doi:10.3389/fimmu.2025.1609871. 40904451 PMC12401910

[63] Narayan VM , Boorjian SA , Alemozaffar M , Konety BR , Shore ND , Gomella LG , et al. Efficacy of intravesical nadofaragene firadenovec for patients with bacillus Calmette-Guérin-Unresponsive nonmuscle-invasive bladder cancer: 5-year follow-up from a phase 3 trial. J Urol. 2024; 212( 1): 74– 86. doi:10.1097/JU.0000000000004020. 38704840 PMC12721637

[64] Bajorin DF , Witjes JA , Gschwend JE , Schenker M , Valderrama BP , Tomita Y , et al. Adjuvant nivolumab versus placebo in muscle-invasive urothelial carcinoma. N Engl J Med. 2021; 384( 22): 2102– 14. doi:10.1056/NEJMoa2034442. 34077643 PMC8215888

[65] Peyrottes A , Ouzaid I , Califano G , Hermieu JF , Xylinas E . Neoadjuvant immunotherapy for muscle-invasive bladder cancer. Medicina. 2021; 57( 8): 769. doi:10.3390/medicina57080769. 34440975 PMC8398505

[66] Suartz CV , de Lima RD , De almeida LS , Liebl B , Lopes RI , Branquinho Reis GB , et al. Neoadjuvant immunotherapy in bladder cancer: Ushering in a new era of treatment—A systematic review of current evidence. Eur Urol Open Sci. 2025; 79: 45– 59. doi:10.1016/j.euros.2025.07.010. 40822997 PMC12351343

[67] Hu J , Chen J , Ou Z , Chen H , Liu Z , Chen M , et al. Neoadjuvant immunotherapy, chemotherapy, and combination therapy in muscle-invasive bladder cancer: A multi-center real-world retrospective study. Cell Rep Med. 2022; 3( 11): 100785. doi:10.1016/j.xcrm.2022.100785. 36265483 PMC9729796

[68] Wu S , Li R , Jiang Y , Yu J , Zheng J , Li Z , et al. Liquid biopsy in urothelial carcinoma: Detection techniques and clinical applications. Biomed Pharmacother. 2023; 165: 115027. doi:10.1016/j.biopha.2023.115027. 37354812

[69] Li S , Xin K , Pan S , Wang Y , Zheng J , Li Z , et al. Blood-based liquid biopsy: Insights into early detection, prediction, and treatment monitoring of bladder cancer. Cell Mol Biol Lett. 2023; 28( 1): 28. doi:10.1186/s11658-023-00442-z. 37016296 PMC10074703

[70] Sivapalan L , Murray JC , Canzoniero JV , Landon B , Jackson J , Scott S , et al. Liquid biopsy approaches to capture tumor evolution and clinical outcomes during cancer immunotherapy. J Immunother Cancer. 2023; 11( 1): e005924. doi:10.1136/jitc-2022-005924. 36657818 PMC9853269

[71] Salehnia Z , Rezaee D , Ehtiati S , Bakhtiari M , Khalilzad MA , Najafi S . Liquid biopsy for the management of gastrointestinal cancers. Clin Chim Acta. 2026; 578: 120474. doi:10.1016/j.cca.2025.120474. 40652998

[72] Chen W , Zhang L , Gao M , Zhang N , Wang R , Liu Y , et al. Role of tertiary lymphoid structures and B cells in clinical immunotherapy of gastric cancer. Front Immunol. 2025; 15: 1519034. doi:10.3389/fimmu.2024.1519034. 39840050 PMC11747648

[73] An D , Chen G , Cheng WY , Mohrs K , Adler C , Gupta NT , et al. LTβR agonism promotes antitumor immune responses via modulation of the tumor microenvironment. Cancer Res. 2024; 84( 23): 3984– 4001. doi:10.1158/0008-5472.CAN-23-2716. 39137402

