# Supplementary material for: Germinal Center–Like Tertiary Lymphoid Structures Mark Immune Responsiveness and Enable Checkpoint Immunotherapy in Bladder Cancer
Source: Oncol Res. 2026 Jun 16;34(7):25. doi: 10.32604/or.2026.077808 (PMC13292039; doi:10.32604/or.2026.077808)
Supplement: Supplementary file 1 [file OncolRes-34-77808-s001.zip › TSP_OR_77808-s001.docx]

**Supplementary Figure S1:** Pro-inflammatory score between GC-like TLS groups. GC: Germinal center; TLS: Tertiary lymphoid structures.

**Supplementary Figure S2:** (A) Functional enrichment of WGCNA modules Brown (ME3) and Yellowgreen (ME36). Upper panels: KEGG (left) and GO-BP (right) enrichment of Brown module; Lower panels: KEGG (left) and GO-BP (right) enrichment of Yellowgreen module. (B) Enrichment score distribution of the intersection gene set across single-cell subtypes. WGCNA: Weighted gene co-expression network analysis; GO: Gene Ontology; BP: Biological Process; KEGG: Kyoto Encyclopedia of Genes and Genomes.

**Supplementary Table S1**

| **Patient** | **Sex** | **Age** | **Grade** | **STAGE** | **pT_stage** | **pN_stage** | **Tumor_Size （mm^3^）** | **Treatment** | **Immunotherapy** | **Responseir ORR** | **TLS_Size (mm^2^)** | **TLS_D** | **M_TLS_D** | **TLS_C** | **M_TLS_C** | **EFS_m** |
| --- | --- | --- | --- | --- | --- | --- | --- | --- | --- | --- | --- | --- | --- | --- | --- | --- |
| **IBC6** | **male** | **63** | **Grade1** | **stage1** | **pT1** | **N0** | **55*49*3** | **ICIs+RC** | **PD-1** | **iPR** | **0.54** | **1.501** | **1.501** | **2** | **2** | **12** |
| **IBC16** | **male** | **60** | **Grade1** | **stage1** | **pT1** | **N0** | **20*20*3** | **ICIs+RC** | **PD-1** | **iSD** | **0.21** | **0.591** | **0** | **3** | **0** | **7** |
| **IBC17** | **male** | **60** | **Grade1** | **stage1** | **pT1** | **N0** | **15*12*3** | **ICIs+RC** | **PD-1** | **iSD** | **0.015** | **0.409** | **0** | **4** | **0** | **6** |
| **IBC18** | **female** | **70** | **Grade1** | **stage1** | **pT1** | **N0** | **30*30*5** | **ICIs+RC** | **PD-1** | **iSD** | **0** | **0** | **0** | **0** | **0** | **8** |
| **IBC22** | **male** | **75** | **Grade1** | **stage1** | **pT1** | **N0** | **25*25*10** | **ICIs+RC** | **PD-1** | **iSD** | **0** | **0** | **0** | **0** | **0** | **6** |
| **IBC24** | **female** | **66** | **Grade1** | **stage1** | **pT1** | **N0** | **25*25*10** | **ICIs+RC** | **PD-1** | **iPR** | **0.529** | **1.471** | **0.817** | **9** | **5** | **14** |
| **IBC27** | **male** | **58** | **Grade1** | **stage1** | **pT1** | **N0** | **15*10*3** | **ICIs+RC** | **PD-1** | **iPR** | **0.756** | **2.101** | **0.84** | **5** | **2** | **14** |
| **IBC29** | **male** | **77** | **Grade1** | **stage1** | **pT1** | **N0** | **90*30*2** | **ICIs+RC** | **PD-1** | **iPR** | **0.884** | **2.456** | **1.474** | **5** | **3** | **12** |
| **IBC31** | **male** | **67** | **Grade1** | **stage1** | **pT1** | **N0** | **40*40*4** | **ICIs+RC** | **PD-1** | **iSD** | **0.77** | **2.139** | **0.713** | **6** | **2** | **13** |
| **IBC37** | **male** | **63** | **Grade1** | **stage1** | **pT1** | **N0** | **20*23*3** | **ICIs+RC** | **PD-1** | **iPR** | **0.945** | **2.625** | **1.969** | **12** | **9** | **11** |
| **IBC38** | **male** | **65** | **Grade1** | **stage1** | **pT1** | **N0** | **20*13*8** | **ICIs+RC** | **PD-1** | **iPR** | **1.304** | **3.621** | **2.414** | **9** | **6** | **16** |
| **IBC40** | **male** | **74** | **Grade1** | **stage1** | **pT1** | **N0** | **20*10*8** | **ICIs+RC** | **PD-1** | **iSD** | **0.968** | **2.689** | **0** | **5** | **0** | **5** |
| **IBC1** | **male** | **63** | **Grade1** | **stage2** | **pT2** | **N0** | **30*30*10** | **ICIs+RC** | **PD-1** | **iPR** | **0.293** | **0.814** | **0.326** | **5** | **2** | **11** |
| **IBC2** | **female** | **52** | **Grade1** | **stage2** | **pT2** | **N0** | **20*10*15** | **ICIs+RC** | **PD-1** | **iSD** | **0** | **0** | **0** | **0** | **0** | **16** |
| **IBC4** | **male** | **59** | **Grade1** | **stage2** | **pT2** | **N0** | **32*36*24** | **ICIs+RC** | **PD-1** | **iPR** | **0.508** | **1.412** | **0.605** | **7** | **3** | **12** |
| **IBC7** | **male** | **72** | **Grade1** | **stage2** | **pT2** | **N0** | **14*26*7** | **ICIs+RC** | **PD-1** | **iSD** | **0** | **0** | **0** | **0** | **0** | **9** |
| **IBC8** | **male** | **53** | **Grade1** | **stage2** | **pT2** | **N0** | **16*24*15** | **ICIs+RC** | **PD-1** | **iSD** | **0.341** | **0.946** | **0.946** | **5** | **5** | **15** |
| **IBC9** | **female** | **64** | **Grade3** | **stage2** | **pT2** | **N0** | **15*18*8** | **ICIs+RC** | **PD-1** | **iPR** | **0.925** | **2.572** | **2.143** | **6** | **5** | **13** |
| **IBC10** | **female** | **70** | **Grade1** | **stage2** | **pT2** | **N0** | **60*26*42** | **ICIs+RC** | **PD-1** | **iSD** | **0** | **0** | **0** | **0** | **0** | **6** |
| **IBC13** | **male** | **61** | **Grade1** | **stage2** | **pT2** | **N0** | **18*15*8** | **ICIs+RC** | **PD-1** | **iSD** | **0.06** | **0.171** | **0.057** | **3** | **1** | **10** |
| **IBC14** | **female** | **58** | **Grade1** | **stage2** | **pT2** | **N0** | **25*50*8** | **ICIs+RC** | **PD-1** | **iSD** | **0.25** | **0.703** | **0.352** | **4** | **2** | **11** |
| **IBC20** | **male** | **70** | **Grade1** | **stage2** | **pT2** | **N0** | **30*15*11** | **ICIs+RC** | **PD-1** | **iSD** | **0.319** | **0.887** | **0** | **3** | **0** | **7** |
| **IBC21** | **male** | **57** | **Grade1** | **stage3** | **pT2** | **N1** | **18*10*15** | **ICIs+RC** | **PD-1** | **iSD** | **0** | **0** | **0** | **0** | **0** | **7** |
| **IBC23** | **male** | **77** | **Grade2** | **stage2** | **pT2** | **N0** | **30*20*5** | **ICIs+RC** | **PD-1** | **iSD** | **0.193** | **0.536** | **0** | **3** | **0** | **9** |
| **IBC25** | **male** | **61** | **Grade1** | **stage2** | **pT2** | **N0** | **30*30*15** | **ICIs+RC** | **PD-1** | **iSD** | **0.203** | **0.565** | **0** | **5** | **0** | **8** |
| **IBC30** | **female** | **70** | **Grade1** | **stage2** | **pT2** | **N0** | **30*20*8** | **ICIs+RC** | **PD-1** | **iPR** | **4.67** | **12.98** | **7.081** | **22** | **12** | **18** |
| **IBC34** | **male** | **59** | **Grade1** | **stage2** | **pT2** | **N0** | **30*20*15** | **ICIs+RC** | **PD-1** | **iSD** | **0.869** | **2.416** | **0** | **7** | **0** | **6** |
| **IBC39** | **male** | **68** | **Grade1** | **stage2** | **pT2** | **N0** | **34*25*8** | **ICIs+RC** | **PD-1** | **iPR** | **1.73** | **4.809** | **3.206** | **21** | **14** | **17** |
| **IBC52** | **female** | **65** | **Grade1** | **stage2** | **pT2** | **N0** | **40*20*25** | **ICIs+RC** | **PD-1** | **iSD** | **0.58** | **0.621** | **0.3105** | **6** | **3** | **11** |
| **IBC53** | **male** | **61** | **Grade1** | **stage2** | **pT2** | **N0** | **40*30*10** | **ICIs+RC** | **PD-1** | **iSD** | **0.48** | **0.75** | **0.214** | **7** | **2** | **12** |
| **IBC54** | **female** | **68** | **Grade1** | **stage2** | **pT2** | **N0** | **35*15*25** | **ICIs+RC** | **PD-1** | **iSD** | **0.36** | **1** | **0.2** | **5** | **1** | **11** |
| **IBC55** | **female** | **65** | **Grade1** | **stage2** | **pT2** | **N0** | **25*35*10** | **ICIs+RC** | **PD-1** | **iPR** | **0.35** | **1.282** | **0.427** | **6** | **2** | **10** |
| **IBC3** | **male** | **72** | **Grade1** | **stage3** | **pT3** | **N1** | **60*58*15** | **ICIs+RC** | **PD-1** | **iSD** | **0.162** | **0.449** | **0.269** | **5** | **3** | **9** |
| **IBC5** | **male** | **61** | **Grade1** | **stage2** | **pT3** | **N0** | **25*20*40** | **ICIs+RC** | **PD-1** | **iPR** | **0.431** | **1.197** | **0.513** | **7** | **3** | **11** |
| **IBC11** | **male** | **67** | **Grade1** | **stage3** | **pT3** | **N1** | **18*17*15** | **ICIs+RC** | **PD-1** | **iSD** | **0.2** | **0.316** | **0.105** | **3** | **1** | **7** |
| **IBC12** | **male** | **71** | **Grade1** | **stage3** | **pT3** | **N1** | **40*18*20** | **ICIs+RC** | **PD-1** | **iPR** | **0.552** | **1.533** | **0** | **5** | **0** | **6** |
| **IBC19** | **male** | **69** | **Grade3** | **stage2** | **pT3** | **N0** | **50*40*30** | **ICIs+RC** | **PD-1** | **iSD** | **0** | **0** | **0** | **0** | **0** | **4** |
| **IBC28** | **male** | **69** | **Grade3** | **stage3** | **pT3** | **N1** | **60*55*12** | **ICIs+RC** | **PD-1** | **iSD** | **0.289** | **0.804** | **0.536** | **3** | **2** | **12** |
| **IBC35** | **female** | **71** | **Grade3** | **stage2** | **pT3** | **N0** | **30*20*15** | **ICIs+RC** | **PD-1** | **iPR** | **1.143** | **3.175** | **0.577** | **11** | **2** | **15** |
| **IBC36** | **male** | **73** | **Grade3** | **stage3** | **pT3** | **N1** | **60*80*20** | **ICIs+RC** | **PD-1** | **iSD** | **0** | **0** | **0** | **0** | **0** | **5** |
| **IBC46** | **female** | **68** | **Grade2** | **stage3** | **pT3** | **N1** | **25*10*20** | **ICIs+RC** | **PD-1** | **iSD** | **0.36** | **1** | **0.25** | **4** | **1** | **10** |
| **IBC47** | **female** | **67** | **Grade3** | **stage3** | **pT3** | **N1** | **30*15*20** | **ICIs+RC** | **PD-1** | **iSD** | **0.55** | **0.655** | **0** | **3** | **0** | **9** |
| **IBC48** | **female** | **69** | **Grade2** | **stage3** | **pT3** | **N1** | **35*15*18** | **ICIs+RC** | **PD-1** | **iSD** | **0.38** | **0.947** | **0** | **1** | **0** | **6** |
| **IBC49** | **female** | **59** | **Grade2** | **stage3** | **pT3** | **N1** | **40*30*10** | **ICIs+RC** | **PD-1** | **iPR** | **0.28** | **1.286** | **0** | **5** | **0** | **9** |
| **IBC50** | **female** | **58** | **Grade3** | **stage3** | **pT3** | **N1** | **35*20*10** | **ICIs+RC** | **PD-1** | **iSD** | **0.85** | **0.426** | **0.071** | **6** | **1** | **7** |
| **IBC51** | **female** | **64** | **Grade3** | **stage3** | **pT3** | **N1** | **18*17*15** | **ICIs+RC** | **PD-1** | **iSD** | **0.68** | **0.529** | **0.106** | **5** | **1** | **6** |
| **IBC26** | **male** | **68** | **Grade3** | **stage4** | **pT4** | **N1** | **30*30*20** | **ICIs+RC** | **PD-1** | **iPR** | **0.783** | **2.175** | **1.088** | **10** | **5** | **13** |
| **IBC32** | **female** | **58** | **Grade3** | **stage4** | **pT4** | **N2** | **40*30*10** | **ICIs+RC** | **PD-1** | **iPD** | **0** | **0** | **0** | **0** | **0** | **5** |
| **IBC33** | **male** | **52** | **Grade3** | **stage4** | **pT4** | **N2** | **50*50*30** | **ICIs+RC** | **PD-1** | **iPR** | **1.982** | **5.505** | **3.503** | **11** | **7** | **13** |
| **IBC41** | **female** | **68** | **Grade3** | **stage4** | **pT4** | **N1** | **55*35*15** | **ICIs+RC** | **PD-1** | **iSD** | **0.102** | **0.282** | **0.094** | **3** | **1** | **5** |
| **IBC42** | **female** | **60** | **Grade3** | **stage4** | **pT4** | **N1** | **45*35*20** | **ICIs+RC** | **PD-1** | **iSD** | **0.38** | **0.947** | **0** | **1** | **0** | **5** |
| **IBC43** | **female** | **58** | **Grade3** | **stage4** | **pT4** | **N1** | **35*45*15** | **ICIs+RC** | **PD-1** | **iSD** | **0.79** | **0.456** | **0.456** | **1** | **1** | **6** |
| **IBC44** | **female** | **59** | **Grade3** | **stage4** | **pT4** | **N1** | **18*17*15** | **ICIs+RC** | **PD-1** | **iSD** | **0.75** | **0.48** | **0** | **2** | **0** | **7** |
| **IBC45** | **female** | **62** | **Grade3** | **stage4** | **pT4** | **N1** | **25*25*15** | **ICIs+RC** | **PD-1** | **iSD** | **0.52** | **0.692** | **0.346** | **2** | **1** | **6** |
| **IBC56** | **female** | **70** | **Grade3** | **stage4** | **pT4** | **N1** | **55*58*15** | **ICIs+RC** | **PD-1** | **iPD** | **0.25** | **0.281** | **0** | **1** | **0** | **5** |
| **IBC57** | **male** | **71** | **Grade3** | **stage4** | **pT4** | **N1** | **35*18*15** | **ICIs+RC** | **PD-1** | **iPD** | **0** | **0** | **0** | **0** | **0** | **4** |
| **IBC58** | **female** | **59** | **Grade3** | **stage4** | **pT4** | **N1** | **20*18*20** | **ICIs+RC** | **PD-1** | **iPD** | **0** | **0** | **0** | **0** | **0** | **7** |
| **IBC59** | **female** | **60** | **Grade3** | **stage4** | **pT4** | **N1** | **35*20*35** | **ICIs+RC** | **PD-1** | **iPD** | **0.58** | **0.238** | **0** | **1** | **0** | **5** |
| **IBC60** | **female** | **65** | **Grade3** | **stage4** | **pT4** | **N1** | **60*35*30** | **ICIs+RC** | **PD-1** | **iPD** | **0.28** | **0.125** | **0** | **1** | **0** | **5** |
| **IBC15** | **male** | **65** | **Grade1** | **stage0** | **pTa** | **N0** | **3*3*1** | **ICIs+RC** | **PD-1** | **iSD** | **0.09** | **0.261** | **0.261** | **1** | **1** | **9** |

**TLS_D: TLS_Density, M_TLS_D: Mature_TLS_Density, TlS_C: TLS_Count, M_TLS_C: Mature_TLS_Conut, EFS_M: EFS_Months, TLS: Tertiary lymphoid structures; EFS: Event-free survival; IBC: Invasive bladder cancer; ICI: Immune checkpoint inhibitor; RC: Radical cystectomy; PD-1:Programmed cell death protein 1; iPD: immune progressive disease; iSD: Immune stable disease; irORR: immune-related Objective Response Rate.**
